# TMT-Based Quantitative Proteomic Analysis Reveals Downregulation of ITGAL and Syk by the Effects of Cycloastragenol in OVA-Induced Asthmatic Mice

**DOI:** 10.1155/2022/6842530

**Published:** 2022-10-25

**Authors:** Xueyi Zhu, Baojun Liu, Zhenhui Ruan, Mengmeng Chen, Congcong Li, Hanlin Shi, Xi Huang, Hang Yu, Yaolong Zhou, Hehua Zhu, Jing Sun, Ying Wei, Weifang Xu, Jingcheng Dong

**Affiliations:** ^1^Department of Integrative Medicine, Huashan Hospital, Fudan University, Shanghai, China; ^2^Institutes of Integrative Medicine, Fudan University, Shanghai, China; ^3^Shenzhen Hospital of Guangzhou University of Chinese Medicine (Futian), Shenzhen, China

## Abstract

**Background:**

Cycloastragenol (CAG) has been reported to alleviate airway inflammation in ovalbumin- (OVA-) induced asthmatic mice. However, its specific mechanisms remain unclear.

**Objective:**

This study is aimed at investigating the effects of CAG on asthma, comparing its efficacy with dexamethasone (DEX), and elucidating the mechanism of CAG's regulation.

**Methods:**

The asthma mouse model was induced by OVA. CAG at the optimal dose of 125 mg/kg was given every day from day 0 for 20-day prevention or from day 14 for a 7-day treatment. We observed the preventive and therapeutic effects of CAG in asthmatic mice by evaluating the airway inflammation, AHR, and mucus secretion. Lung proteins were used for TMT-based quantitative proteomic analysis to enunciate its regulatory mechanisms.

**Results:**

The early administration of 125 mg/kg CAG before asthma happened prevented asthmatic mice from AHR, airway inflammation, and mucus hypersecretion, returning to nearly the original baseline. Alternatively, the administration of CAG during asthma also had the same therapeutic effects as DEX. The proteomic analysis revealed that the therapeutical effects of CAG were associated with 248 differentially expressed proteins and 3 enriched KEGG pathways. We then focused on 3 differentially expressed proteins (ITGAL, Syk, and Vav1) and demonstrated that CAG treatment downregulated ITGAL, Syk, and Vav1 by quantitative real-time PCR, western blot analysis, and immunohistochemical staining.

**Conclusion:**

These findings suggest that CAG exerts preventive and protective effects on asthma by inhibiting ITGAL, Syk, and the downstream target Vav1.

## 1. Introduction

Asthma, triggered by gene-environment factors, is often characterized by airway hyperresponsiveness (AHR), inflammation, and remodeling [1]. Its typical symptoms include wheezing, shortness of breath, chest tightness, and coughing [2]. Currently, oral dexamethasone (DEX) is used for acute asthma as the inhaled corticosteroids (ICS)/long-acting beta2-agonist (LABA) is used for chronic asthma [3, 4]. However, these corticosteroids cause several side effects, such as osteoporosis, obesity, and drug resistance [5]. Besides, emerging targeted biologic therapies, such as omalizumab (anti-IgE) and mepolizumab (anti-IL-5), have generated a large economic burden and are of no use in the prevention of virus infection-induced asthma [6]. Thus, seeking a novel therapeutic better than these we already have for asthma treatment and clarifying the specific targets remain urgent.

Some recent studies have investigated the regulatory effect of active ingredients from traditional Chinese medicine on airway inflammation in asthma, mainly including polyphenols, flavonoids, alkaloids, and terpenoids [7]. For example, curcumin (which belongs to polyphenols) is proven to reduce airway inflammation, mucus secretion, and AHR in asthmatic mice by blocking NF-*κ*B, downregulating Notch, and activating Nrf2/HO-1 pathways [8–10]. It has been found that icariin, as a member of the flavonoid family, can regulate both Th1/Th2 and Th17/Treg balance in the asthmatic model [11, 12]. Also, ligustrazine (alkaloids) has the potential to alleviate allergic airway inflammation in asthmatic patients and rats with the reduction of eosinophils and neutrophils [13, 14]. As an important terpenoid, Astragaloside IV (AST) is the main component of the pharmacologic activity of Astragalus membranaceus (Huang-Qi), which is regarded as an effective herb for asthma in traditional Chinese medicine [15]. It has been reported in many articles that the anti-inflammatory effect of AST in the process of asthma is mainly associated with the regulation of NF-*κ*B, mTORC1, MAPK, JAK2/STAT6, and Nrf2/HO-1 signaling pathways [7, 16, 17].

Cycloastragenol (CAG) is a bioactive metabolite of AST, with a far higher oral bioavailability than it [18]. The study showed that CAG could inhibit apoptosis and neuroinflammation, which was probably related to its capability of inhibiting the expression of proinflammatory cytokines in the brain such as TNF-*α* and IL-1*β* and upregulating the expression of SIRT1 [19]. Another research explored its effect on abdominal aortic aneurysm (AAA), finding that CAG plays a protective role in AAA progression by inhibiting the activity of MMP-2, suppressing calcification, and protecting elastin [20]. Moreover, CAG targeted NLRP3 inflammasome and regulated skin inflammation in the imiquimod- (IMQ-) induced psoriasiform dermatitis mouse model [21]. Nevertheless, although dozens of articles have discussed the protective effects of CAG in various diseases, few referred to its effect on asthma, with the specific mechanisms remaining unclear. Hence, in this study, we validated the preventive and therapeutic effects of CAG in asthma and attempted to find the specific targets regulated by CAG via TMT-based quantitative proteomic analysis. Given our previous finding that 125 mg/kg of CAG was the optimal dose in asthmatic mice [22], we use it as a dosage in this study. We hope to develop CAG as a novel and safe food source therapeutic for asthma in the future and make it become albumin-based nanomedicines.

## 2. Materials and Methods

### 2.1. Animals

BALB/c female mice (6-8 weeks) weighing 20-25 g were provided by the Jiesijie Laboratory Animal Co., Ltd. (license number: SYXK (Hu) 2020-0032, Shanghai, China). Mice were housed in specific pathogen-free conditions at 22 ± 2°C with 50% humidity and exposed to sterilization feed for mice and water. The animal care and experimental procedures were approved by the Animal Care and Use Committee of Fudan University (Approval No. 2018-10-HSYY-DJC-01).

### 2.2. OVA-Induced Asthma Model Establishment

Mice were randomly divided into the normal control (N) group, OVA-induced asthma model (A) group, CAG prevention (CP) group, CAG treatment (CT) group, and DEX (D) group (6 mice per group). The latter four groups were sensitized on days 0 and 7 by intraperitoneal injection of OVA (100 *μ*g/mouse, grade V, Sigma-Aldrich, St. Louis, MO) combined with aluminum hydroxide (10 mg, Thermo Scientific). After 7 days, they were challenged by aerosol inhalation of 3% OVA once per day for 7 consecutive days. Simultaneously, the N group was sensitized and challenged by PBS instead.

### 2.3. Treatment

**Figure 1:**
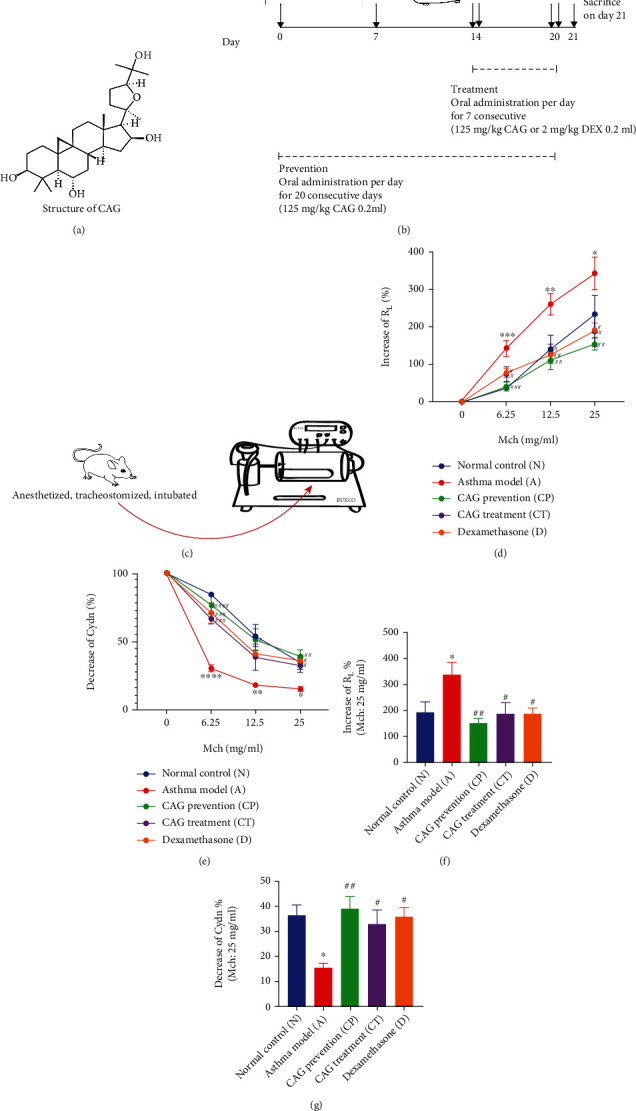
CAG returned *R*_L_ and Cdyn to the baseline in OVA-induced murine asthma model. (a) Molecular structure of CAG. (b) Induction of asthmatic mice and therapeutic schemes. (c) Flowchart of AHR measurement. Changes in (d) *R*_L_ and (e) Cdyn at increased doses of Mch. Changes in (f) *R*_L_ and (g) Cdyn at 25 mg/ml Mch. *n* = 6 in each group of the normal control (N) group, OVA-induced asthma model (A) group, CAG prevention (CP) group, CAG treatment (CT) group, and dexamethasone (D) group. Error bars are means ± SEMs. ^∗^*P* < 0.05, ^∗∗^*P* < 0.01, ^∗∗∗^*P* < 0.001, and ^∗∗∗∗^*P* < 0.0001 vs. N group; #*P* < 0.05, ##*P* < 0.01, ###*P* < 0.001, and ####*P* < 0.0001 vs. A group by one-way ANOVA.

Cycloastragenol (CAG, purity > 98%) was obtained from a commercial source (Winherb Medical Science Co., Ltd., Shanghai, CHN) (Figure 1(a)). The CP group received 125 mg·kg^−1^ CAG [23] once a day since sensitization started. As aerosol inhalation began, the CT group and D group, respectively, received 125 mg·kg^−1^ CAG [23] and 2 mg·kg^−1^ DEX [24] once a day by intragastric administration. All mice were sacrificed 24 h after the last administration. The protocol is in Figure 1(b).

### 2.4. Measurement of Airway Hyperresponsiveness (AHR)

Mice were anesthetized by intraperitoneal injection with 2% phenobarbital sodium (50 mg/kg) intraperitoneally. Then, mice were tracheostomized, intubated, and connected to the pneumotach, ventilator, and nebulizer (DSI, Buxco Electronics) (Figure 1(c)). Before each sacrifice, total lung resistance (*R*_L_) and dynamic lung compliance (Cdyn) in response to aerosolized methacholine (0, 6.25, 12.5, and 25 mg/ml Mch, Sigma-Aldrich, St. Louis, MO) were measured by whole-body plethysmograph with a single-chamber (FinePointe RC System, DSI Buxco Electronics). Data were acquired and analyzed by FinePointe™ data acquisition and analysis software (DSI, Buxco Electronics, NY, USA).

### 2.5. Leukocyte Classification and Counts of Bronchoalveolar Lavage Fluid (BALF)

BALF was collected by endotracheal intubation with 300 *μ*l ice-cold PBS twice and then centrifuged at 500*g* for 10 min at 4°C. The supernatants were used for further ELISA. The total cells were resuspended with 50 *μ*l PBS and counted via the Mindray BC-5000Vet automated hematology analyzer (Mindray, Shenzhen, CHN).

### 2.6. ELISA

Blood serum was centrifuged at 500*g* for 25 min at 24°C. The supernatant was collected and detected by ELISA kits of immunoglobulin E (IgE) (MultiSciences), ALT, AST, TBIL, Cr (Lengton, Shanghai, China), and BUN (Njjcbio, Nanjing, China). The amounts of interleukin- (IL-) 4, IL-5, IL-13, and IL-17A of BALF supernatant were determined by ELISA kits (MultiSciences, Hangzhou, China) following the manufacturer's instruction.

### 2.7. Histological Analysis

Lung sections of the middle lobe of the left lung from all groups were fixed in formalin, embedded in paraffin, and then cut into 4 *μ*m sections. Hematoxylin-eosin (H&E) staining [25] and periodic acid-Schiff (PAS) staining [26] were performed to analyze the inflammation of the trachea and evaluate the secretion of mucus. The left liver and kidneys from the N group, CP group, and CT group were fixed and embedded the same as the steps of lung histology. H&E staining, together with the amounts of ALT, AST, TBIL, Cr, and BUN, was used to assess the liver and kidney toxicity of 125 mg/kg CAG.

### 2.8. TMT-Based Quantitative Proteomic Analysis

Lung tissues (the lower lobe of the right lung) were homogenized in lysis buffer to extract proteins. Protein concentration was quantified using a BCA kit (Thermo Scientific, USA). Then, the protein solutions were digested with trypsin, labeled using a TMT kit (Thermo Scientific, USA), and fractionated via an 1100 HPLC System (Agilent) with an Agilent Zorbax Extend RP column. The peptides were separated into 15 fractions, dried by vacuum centrifugation, and followed by LC-MS/MS for analysis. The resulting MS/MS data were processed using Proteome Discover 2.4 (Thermo Fisher, USA). Tandem mass spectra were searched against the Swiss-Prot Mouse knowledgebase (https://www.uniprot.org/) concatenated with the reverse decoy database. Trypsin/P was specified as a cleavage enzyme allowing up to 2 missing cleavages. The mass tolerance for precursor ions was set to 10 ppm as well as the mass tolerance for fragment ions was set to 0.02 Da. Carbamidomethyl on Cys was specified as a fixed modification. Oxidation on methionine and acetylation of the protein N-terminal were specified as variable modifications. The TMT-10plex was set as a quantitative method. Protein groups considered for quantification required at least 2 peptides (1 unique peptide at least), and the global false discovery rate (FDR) was set to 1%. After that, the differentially expressed proteins (DEPs) were identified according to fold change > 1.2 or fold change < 0.83 and *P* value < 0.05. DEPs were further analyzed using Gene Ontology (GO) knowledgebase (http://geneontology.org/) for functional analysis. Meanwhile, DEPs were mapped to the KEGG database (http://www. genome.jp/kegg/). A hypergeometric distribution test was applied to determine the significance of the enriched GO term or KEGG pathway. The formula for calculating the *P* value by hypergeometric distribution test was as follows:

(1) $$\begin{array}{c} P=1-\overset{m-1}{\underset{i=0}{\sum }}\frac{\left(\begin{array}{c} M\\ i \end{array}\right)\left(\begin{array}{c} N-M\\ n-i \end{array}\right)}{\left(\begin{array}{c} N\\ n \end{array}\right)}. \end{array}$$

### 2.9. Quantitative Real-Time PCR

Total RNA was isolated from lung tissues using the Total RNA Extraction Mini Kit (ONREW, Guangdong, China). After evaluating RNA concentration and quality, the extracted RNA was reversely transcribed into cDNA using PrimeScript™RT Master Mix (Perfect Real Time) (Takara Biomedical Technology Co., Ltd., Beijing, China). The primer sequences are listed in Table 1. And relative gene expressions were quantified by Genious 2X SYBR Green Fast qPCR Mix (Low ROX Premixed) (ABclonal, Wuhan, China) with QuantStudio™ 6 Flex Real-Time PCR System (Thermo Fisher Scientific, USA). The level of mRNAs was calculated using the 2^-*ΔΔ*Ct^ method and presented as a ratio to *β*-actin.

### 2.10. Western Blot Analysis

Lung tissues were minced and lysed in ice-cold RIPA Lysis Buffer containing phosphatase inhibitors and a protease inhibitor to obtain protein. Protein concentrations were quantified with Pierce BCA Protein Assay Kit (Thermo Scientific). For western blot, 30 *μ*g of protein was loaded in each well and separated by 10% SDS-PAGE, and then, the protein bands were electro-transferred onto 0.2 *μ*m PVDF membranes by the eBlot™ L1 wet protein transfer system (GenScript). The blocked blots were incubated with anti-CD11*α* (anti-ITGAL, 1 : 1000, ab228964, Abcam), anti-Syk (1 : 1000, 13198T, Cell Signaling Technology), anti-Vav1 (1 : 1000, ab97574, Abcam), and anti-beta actin (1 : 3000, AF7018, Affinity) at 4°C overnight, followed by incubation with HRP-conjugated secondary antibodies (1 : 10000) for 1 h. The blots were visualized using ImageQuant LAS-4000 mini (Fujifilm Corporation, Tokyo, JP) and then quantified by the ImageJ software.

### 2.11. Immunohistochemical Staining

Lung slides were dewaxed, rehydrated, and blocked with 3% bovine serum albumin (BSA) for antigen retrieval. Then, the slides were incubated with anti-ITGAL (1 : 4000), anti-Syk (1 : 300), and anti-Vav1 (1 : 200) overnight at 4°C, followed by incubation with HRP-conjugated secondary antibodies (1 : 200) for 1 h. The slides were visualized using a microscope (×100). Five random sights were selected and analyzed by the ImageJ software.

### 2.12. Molecular Docking Simulation

The 3D structure of CAG was acquired from the PubChem database (https://pubchem.ncbi.nlm.nih.gov/, PubChem CID: 13943286) [27]. Then, ITGAL, Syk, and Vav1 structures were downloaded from RCSB Protein Data Bank (PDB, https://www.rcsb.org/) [28]. Next, AutoDockTools 1.5.6 was applied to remove water molecules and add polar hydrogen atoms [29]. With the setting of the coordinates of the target active pocket, molecular docking was simulated by AutoDock Vina [30]. Finally, the highest scored docking results were visualized by PyMoL 2.4.0 [31].

### 2.13. Statistical Analysis

The data were expressed as means ± SEMs and analyzed by one-way analysis of variance (ANOVA) followed by Tukey's multiple comparison test with GraphPad Prism 8. *P* value < 0.05 was considered significant.

## 3. Results

### 3.1. CAG Ameliorated AHR in OVA-Induced Murine Asthma Model

With exposure to OVA, *R*_L_ was increased, and Cdyn was decreased significantly (*P* at least < 0.05) in response to 6.25, 12.5, and 25 mg/ml Mch but was rapidly reverted by both CAG prevention and treatment (Figures 1(d) and 1(e)). Specifically, at a dose of 25 mg/ml Mch, CAG prevention (*P* < 0.01), CAG treatment (*P* < 0.05), and DEX treatment (*P* < 0.05) all sharply returned the *R*_L_ and Cdyn to the baseline (Figures 1(f) and 1(g)). The results reflected that CAG administration effectively attenuated AHR in the development of asthma, and its therapeutic effect was comparable to DEX.

### 3.2. CAG Decreased Inflammatory Cell Infiltration and Mucus Hypersecretion

**Figure 2:**
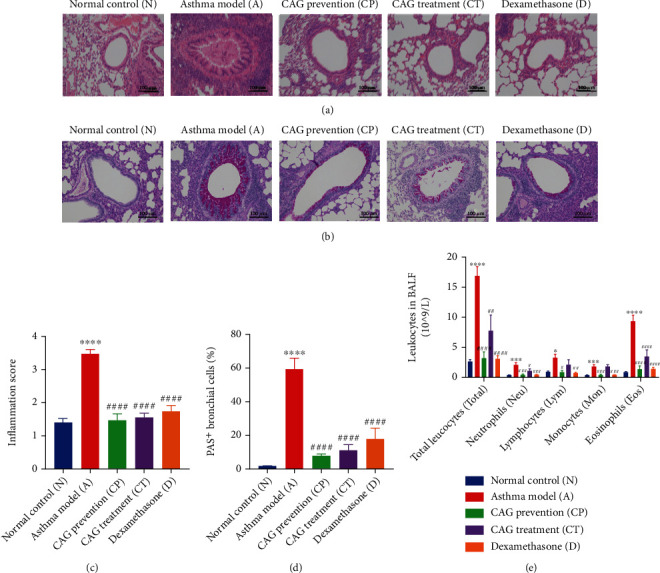
CAG eliminated OVA-induced lymphocytic aggregations and mucus hypersecretion. (a) Representative images of H&E staining and (b) PAS staining of lung sections 24 h after mice sacrifice (×100). Scale bar: 100 *μ*m. (c) Inflammation score with H&E staining. (d) Percentage of PAS^+^ bronchial cells. (e) Counts of total leucocytes (Total), neutrophils (Neu), lymphocytes (Lym), monocytes (Mon), and eosinophils (Eos) in BALF. *n* = 6 in each group of the normal control (N) group, OVA-induced asthma model (A) group, CAG prevention (CP) group, CAG treatment (CT) group, and dexamethasone (D) group. Error bars are means ± SEMs. ^∗^*P* < 0.05, ^∗∗∗^*P* < 0.001, and ^∗∗∗∗^*P* < 0.0001 vs. N group; #*P* < 0.05, ##*P* < 0.01, ###*P* < 0.001, and ####*P* < 0.0001 vs. A group by one-way ANOVA.

CAG pretreatment and treatment, similar to DEX treatment, repaired OVA-boosted infiltration of inflammatory cells and hypersecretion of mucus around the airways (Figures 2(a) and 2(b)). According to the inflammation score analyzed by H&E staining and the percentage of PAS^+^ bronchial cells acquired by PAS staining, inflammatory cell infiltration, and mucus secretion were extremely excessive in the A group (*P* < 0.0001) compared to the N group. Of note, CP, CT, and D groups all reverted from these hyperinflammatory states (*P* < 0.0001) (Figures 2(c) and 2(d)). Meanwhile, OVA remarkably enhanced total leucocytes (Total), neutrophils (Neu), lymphocytes (Lym), monocytes (Mon), and eosinophils (Eos) (*P* at least < 0.05). As expected, CP, CT, and D groups robustly diminished all these levels (*P* at least < 0.05) but no statistical difference of Lym and Mon in the CT group (Figure 2(e)). The results indicated that CAG administration effectively blocked high inflammation status and excessive mucus secretion in the development of asthma, which was in line with the therapeutic effects of DEX.

### 3.3. CAG Alleviated Inflammatory Cytokines and IgE without Liver or Kidney Toxicity

**Figure 3:**
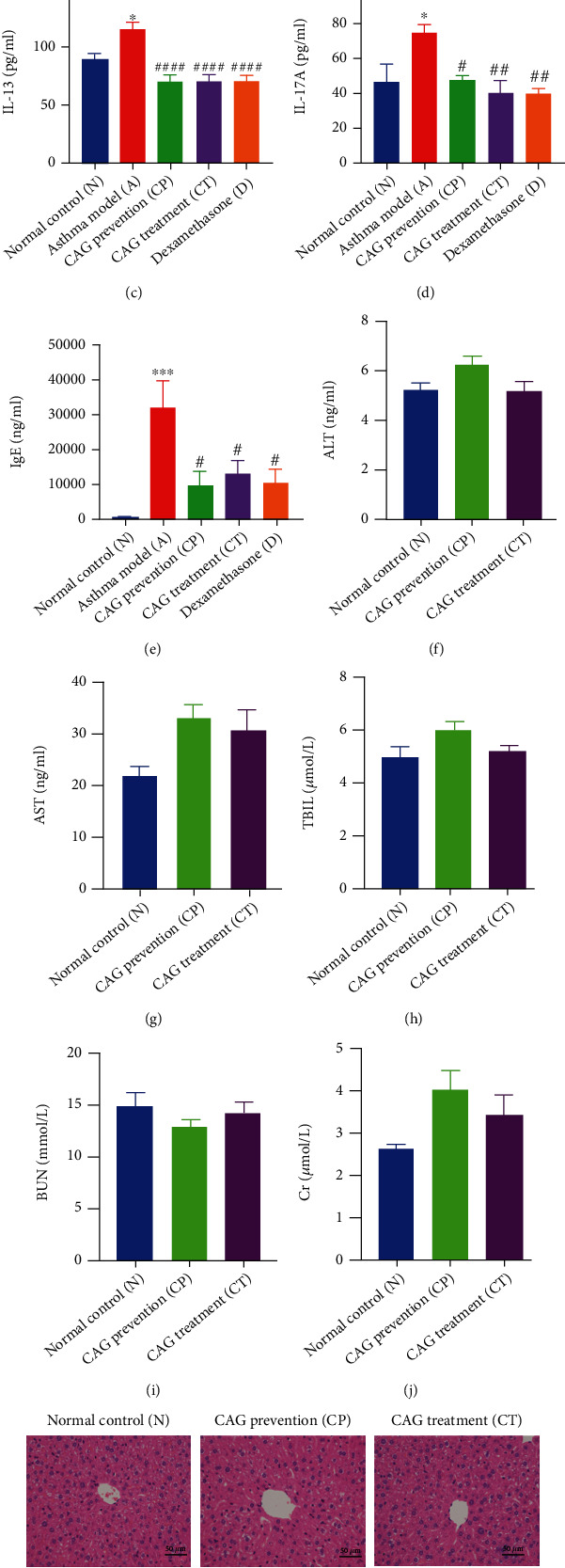
CAG inhibited proinflammatory cytokines and IgE without liver or kidney toxicity. (a) The expression of BALF IL-4. (b) The expression of BALF IL-5. (c) The expression of BALF IL-13. (d) The expression of BALF IL-17A. (e) The expression of serum IgE. (f) The expression of serum ALT. (g) The expression of serum AST. (h) The expression of serum TBIL. (i) The expression of serum BUN. (j) The expression of serum Cr. (k) Representative images of liver sections and kidney sections (l) via H&E staining (×200). Scale bar: 50 *μ*m. *n* = 6 in each group of the normal control (N) group, OVA-induced asthma model (A) group, CAG prevention (CP) group, CAG treatment (CT) group, and dexamethasone (D) group. Error bars are means ± SEMs. ^∗^*P* < 0.05, ^∗∗^*P* < 0.01, and ^∗∗∗^*P* < 0.001 vs. N group; #*P* < 0.05, ##*P* < 0.01, ###*P* < 0.001, and ####*P* < 0.0001 vs. A group by one-way ANOVA.

T helper (Th) 2 and Th17-associated cytokines were dominant in the pathogenesis of asthma [32]. Besides, IgE acted as a crucial player in the allergy response [33]. We observed that the levels of IL-4, IL-5, IL-13, IL-17, and IgE of the A group were grown up. In comparison with the A group, the mice in CP, CT, and D groups had similar remarkable decreases (*P* at least < 0.05) (Figures 3(a)–3(e)). To figure out whether CAG pretreatment and treatment had liver and kidney toxicity, we detected related indexes. ALT, AST, TBIL, BUN, and Cr were close in N, CP, and CT groups. However, AST and Cr were increased slightly in the CP group without a statistical difference (Figures 3(f)–3(j)). Furthermore, compared with the N group, the results of H&E staining of liver sections and kidney sections showed no significant difference in both CP and CT groups (Figures 3(k) and 3(l)). The results implied that CAG administration effectively relieved abundant proinflammatory cytokines, not causing liver and kidney toxicity.

### 3.4. TMT-Based Quantitative Proteomic Analysis of Lung Tissues

**Figure 4:**
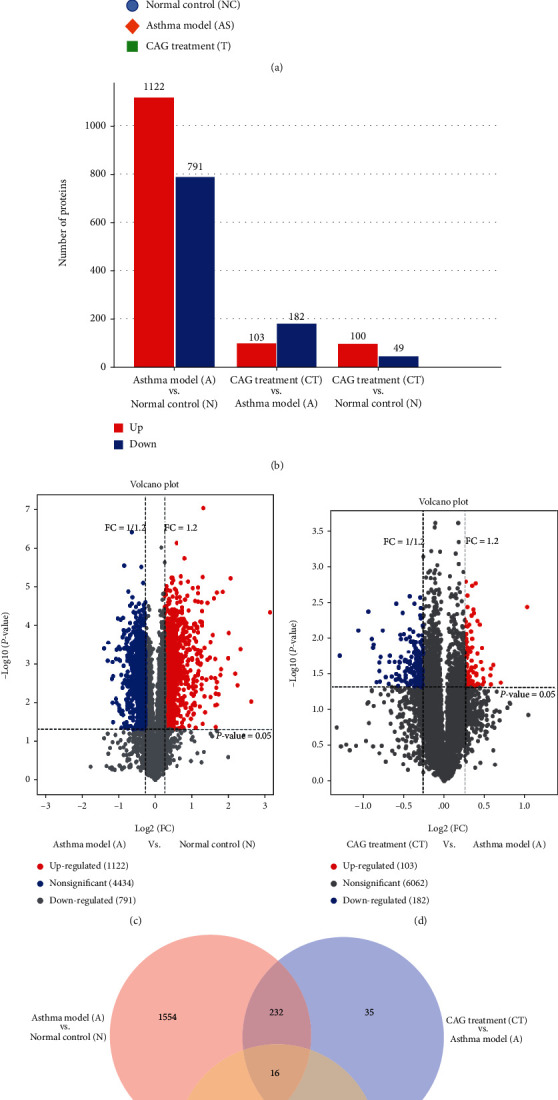
Proteins of lung tissues in the N, A, and CT groups analyzed by TMT-based quantitative proteomic analysis. (a) Principal component analysis (PCA) of N, A, and CT groups based on identified proteins. (b) The number of significantly changed proteins in A vs. N, CT vs. A, and CT vs. N. (c, d) Volcano plot of identified proteins (especially significantly changed proteins) in A vs. N and CT vs. A. Red and blue indicated significantly upregulated or downregulated proteins. (e) Venn diagram of significantly changed proteins and their overlapped proteins (the overlapping of the red circle and blue circle represented 248 overlapped DEPs regulated by CAG). *n* = 3 in each group of the normal control (N/NC) group, OVA-induced asthma model (A/AS) group, and CAG treatment (CT/T) group.

To explore the therapeutic effects for asthma of CAG, lung tissues of N, A, and CT groups were utilized for TMT-based quantitative proteomic analysis. Principal component analysis (PCA) showed that the A group separated from the N group, partly reversed by CAG treatment (Figure 4(a)). Proteomic analysis identified that OVA induction significantly upregulated 1122 proteins and downregulated 791 proteins compared to the N group. However, compared to the A group, CAG treatment caused a significant change of 285 (103 upregulated and 182 downregulated) proteins. Alternatively, compared to the N group, CAG administration resulted in 149 significantly changed (100 upregulated and 49 downregulated) proteins (Figures 4(b)–4(d)). Notably, 248 overlapped DEPs directly regulated by CAG were identified (Figure 4(e)).

**Figure 5:**
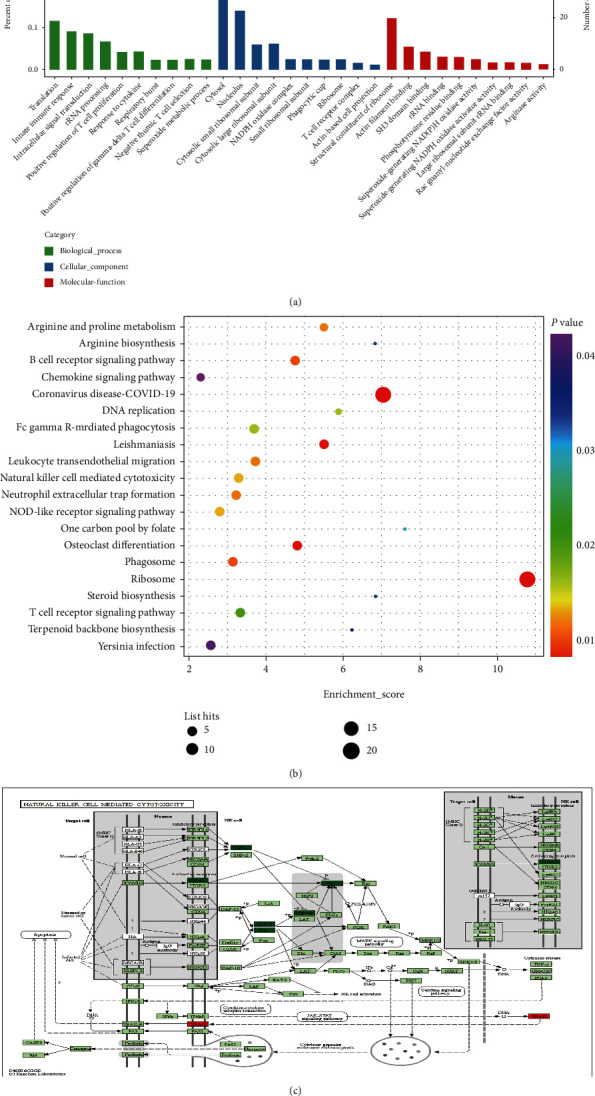
Bioinformatics analysis of 248 DEPs regulated by CAG in OVA-induced murine asthma model. (a) Functional categorization of DEPs using GO knowledgebase. (b) KEGG pathway analysis of DEPs. (c) KEGG pathway map of natural killer cell-mediated cytotoxicity (mmu04650) (dark green and red indicated downregulated or upregulated DEPs by CAG). *n* = 3 in each group.

Based on these 248 DEPs (Table S1), we performed Gene Ontology (GO) and Kyoto Encyclopedia of Genes and Genomes (KEGG) pathway enrichment analyses. The results of the top 30 GO terms revealed that the biological process (BP), cellular component (CC), and molecular function (MF) of DEPs were mainly related to the functions of NADPH oxidase and T cells (Figure 5(a)). Of importance, KEGG enrichment analysis demonstrated that DEPs were dominantly correlated with pathways, including natural killer cell-mediated cytotoxicity (mmu04650), leukocyte transendothelial migration (mmu04670), B cell receptor signaling pathway (mmu04662), and T cell receptor signaling pathway (mmu04064) (Figure 5(b)). Then, we focused on 3 immune-related DEPs of ITGAL, Syk, and Vav1, which have the potential to modulate T, NK, and B cells (Figure 5(c)). These results suggested that 3 DEPs of ITGAL, Syk, and Vav1 might be the therapeutic targets of CAG in the development of asthma (Table 2).

### 3.5. CAG Suppressed ITGAL, Syk, and Vav1 in Lung Tissues, Probably via the Inhibition of p38 MAPK Signaling in Lung Tissues

**Figure 6:**
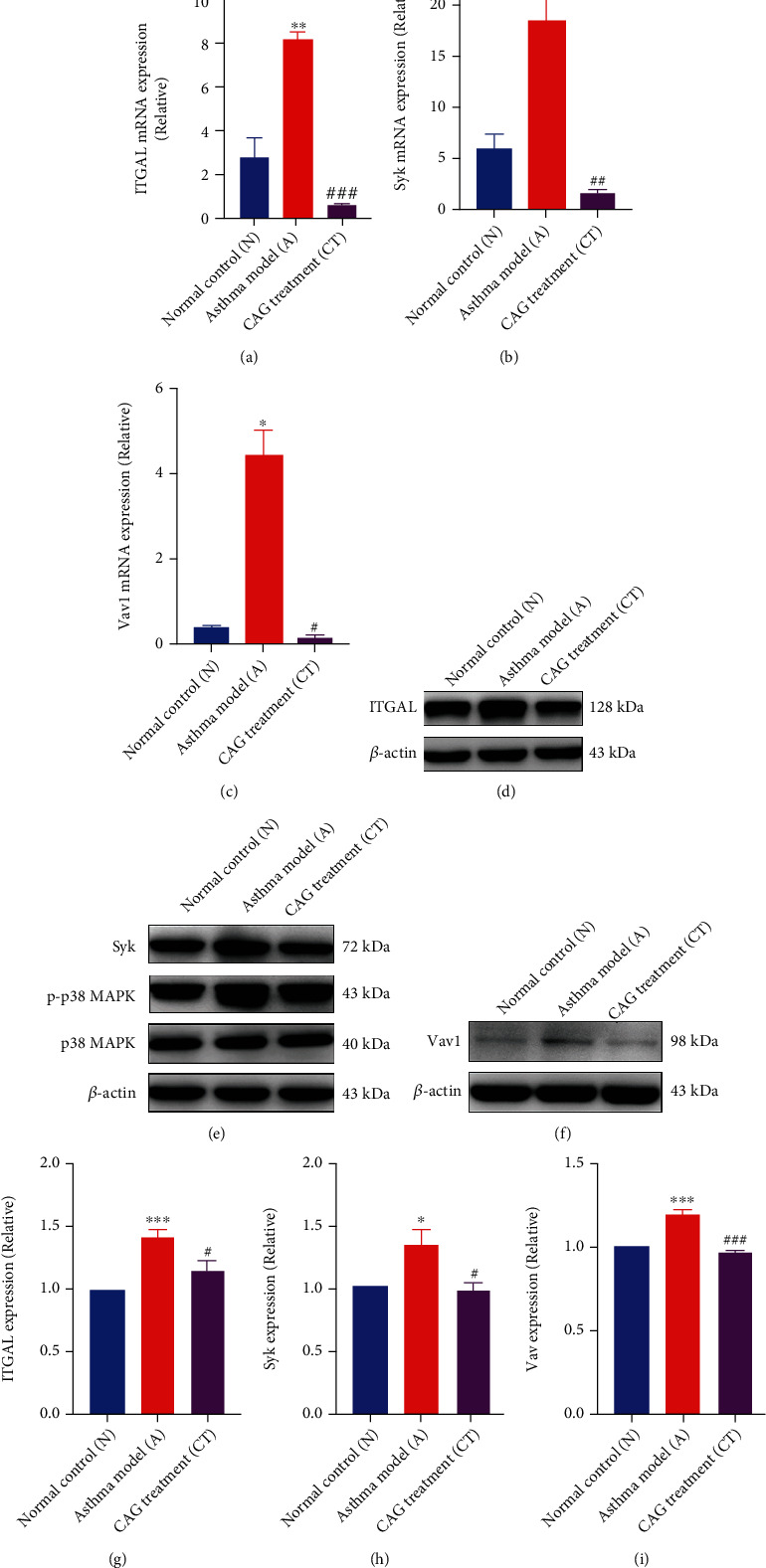
CAG repressed the upstream targets ITGAL and Syk and the downstream target Vav1, probably via p38 signaling in lung tissues (verified by qRT-PCR and western blot). (a) The expression of ITGAL mRNA. (b) The expression of Syk mRNA. (c) The expression of Vav1mRNA. (d) Protein expression of ITGAL. (e) Protein expression of Syk, p-p38 MAPK, and p38 MAPK. (f) Protein expression of Vav1. (g) Relative quantification of ITGAL. (h) Relative quantification of Syk. (i) Relative quantification of Vav1. *n* = 6 in each group of the normal control (N) group, OVA-induced asthma model (A) group, and CAG treatment (CT) group. Error bars are means ± SEMs. ^∗^*P* < 0.05, ^∗∗^*P* < 0.01, ^∗∗∗^*P* < 0.001, and ^∗∗∗∗^*P* < 0.0001 vs. N group; #*P* < 0.05, ##*P* < 0.01, ###*P* < 0.001, and ####*P* < 0.0001 vs. A group by one-way ANOVA.

**Figure 7:**
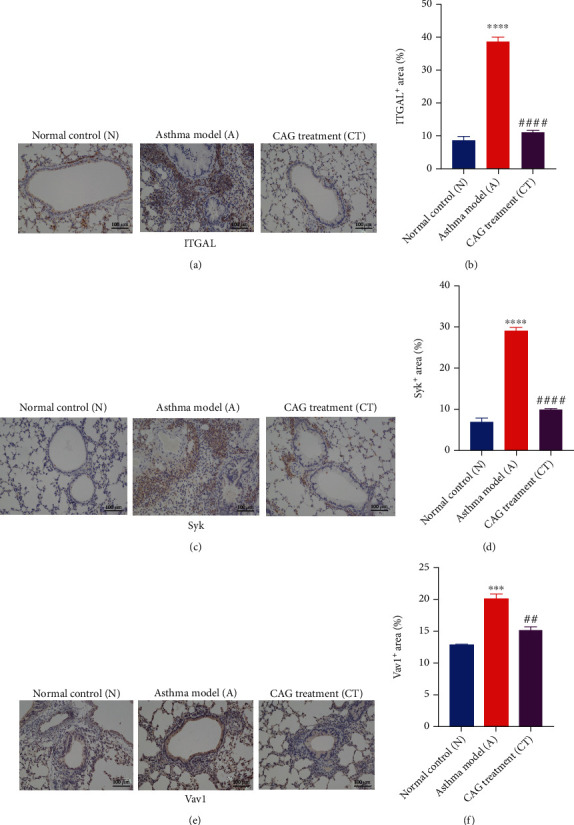
CAG declined ITGAL, Syk, and Vav1 proteins in lung tissues (verified by immunohistochemical staining, ×100, scale bar: 100 *μ*m). (a) Immunohistochemistry examinations of ITGAL. (b) Positive expression of ITGAL. (c) Immunohistochemistry examinations of Syk. (d) Positive expression of Syk. (e) Immunohistochemistry examinations of Vav1. (f) Positive expression of Vav1. *n* = 6 in each group of the normal control (N) group, OVA-induced asthma model (A) group, and CAG treatment (CT) group. Error bars are means ± SEMs. ^∗∗^*P* < 0.01, ^∗∗∗^*P* < 0.001, and ^∗∗∗∗^*P* < 0.0001 vs. N group; #*P* < 0.05, ##*P* < 0.01, and ####*P* < 0.0001 vs. A group by one-way ANOVA.

To further validate the proteomic results, we examined both the mRNA expression and protein expression of the key DEPs (ITGAL, Syk, and Vav1). Although OVA induction markedly upregulated ITGAL, Syk, and Vav1 mRNAs (*P* at least < 0.05), the expression of these mRNAs was returned to normal levels by CAG treatment (*P* at least < 0.05) (Figures 6(a)–6(c)). Besides, the results of western blot and immunohistochemistry were consistent with the results of qRT-PCR. Compared to the N group, the protein expression of ITGAL, Syk, and Vav1 was dramatically lifted in the A group (*P* at least < 0.05) while CAG treatment caused a prominent diminish in these proteins (*P* at least < 0.05) (Figures 6(d)–6(i) and 7(a)–7(f)). Furthermore, we dug out whether the MAPK signaling pathway enriched in our results of KEGG enrichment was involved in this course. We observed that OVA administration significantly activated p38 MAPK, inhibited by CAG. Taken together, CAG administration effectively downregulated the upstream targets ITGAL and Syk and blocked the downstream target Vav1, probably via p38 MAPK signaling.

## 4. Discussion

In this study, we used an asthma mouse model induced by OVA to investigate the preventive and therapeutic effects of CAG. CAG was administrated from the beginning of sensitization or challenge for 14 or 7 days. Our results revealed that CAG could prevent the formation of asthma and reverse the formed asthma. Here, we provide adequate evidence of the preventive and therapeutic effects of CAG in asthma formation and development. Besides, it is the first clue of the specific mechanisms to illustrate how CAG functions as an anti-inflammatory agent of asthma through TMT-based proteomics.

The significant features of asthma are AHR, airway inflammation, and mucus secretion [34]. AHR results from exaggerated airway stenosis with constriction of the airway smooth muscle (ASM) or airway blockage [35]. Airway inflammation is often accompanied by type 2 and type 17 immune responses. Th2-associated cytokines IL-4, IL-13, and IL-5 mediate B cells to generate IgE or accelerate eosinophil recruitment and release, thus causing AHR and mucus secretion [36]. Additionally, Th17-associated IL-17A is dominant in neutrophilic asthma, and its excessive accumulation leads to asthma aggravation [37, 38]. In the development of asthma, airway goblet cell hyperplasia and hypersecretion are boosted to form airway mucus plugs [39]. In our research, CAG declined AHR and alleviated the immune cell abundance in the airway. It also diminished the proinflammatory cytokines mentioned above remarkably and greatly reverted the mucus hypersecretion. To our satisfaction, no liver and kidney toxicity was found in CAG prevention and treatment, which implied the possibility for CAG to be a novel drug for asthma treatment. Therefore, we further explored the specific mechanisms and targets via TMT-based proteomic analysis. The analysis revealed that CAG markedly regulated 248 (164 downregulated and 84 upregulated) DEPs. Based on these results, we focused that CAG might modulate the core signaling pathways (natural killer cell-mediated cytotoxicity, leukocyte transendothelial migration, B cell receptor signaling pathway, and T cell receptor signaling pathway) in asthma pathogenesis. Meanwhile, CAG might significantly regulate the signaling pathway-related DEPs (ITGAL, Syk, and Vav1), which are closely related to T cell regulation.

As the targets proteomic analysis located, ITGAL, Syk, and Vav1 remain crucial in the immune regulation of airway inflammation in asthma, particularly T cell regulation. Integrin alpha L (ITGAL), also known as CD11a, forms intercellular adhesion molecule-1 (ICAM-1) with CD11b and the ICAM-1 receptor lymphocyte function-associated antigen-1 (LFA-1) with CD18, mediating leukocyte adhesion and migration in the development of inflammatory lesions [40–42]. It has the potential to recruit immune cells (such as T cells, neutrophils, and NK cells) to lymphoid tissues, which is essential for antigen-dependent immune activation [43]. ITGAL stimulates T cell receptors and influences T cell adhesion [44]. Moreover, ITGAL modulates the crosstalk between circulating leukocytes and epithelial cells to affect cell adhesion and migration to develop the inflammation of the epithelium [45]. ITGAL plays an important role in Th2 cell homing [41, 46]. Research showed that Th2-dominant allergic airway inflammation was downregulated in mice deficient in CD11a or administrated with an inhibitor of LFA-1 [47]. Gupta et al. [48] observed that compared to healthy controls, the expression of ITGAL was significantly higher in peripheral blood mononuclear cells (PBMCs) in asthmatic patients. Currently, anti-ITGAL antibodies, such as efalizumab, have been reported to be well-established in autoimmune diseases [49].

Another target spleen tyrosine kinase (Syk) is a key kinase in the signal transformation of adaptive immune receptors, which can enhance the affinity of integrin receptors for their ligands. For example, T cell receptors are stimulated for T cell activation and development by binding to integrin [50]. As an upstream regulatory factor of activation of T cells, Syk promotes TCR signaling cascade to initiate and amplify TCR signaling [51]. As a promising therapeutic target for acute and chronic asthma, it is correlated with Th2-dominant airway inflammation, AHR, and pulmonary collagen deposition. Studies demonstrated that Syk inhibition could reverse these pathological states [52]. Syk also has the potential to bind to ITGAL to develop antigen recognition of T cells and the production of IgE by B cells and mast cells [53–55]. Syk is also located upstream of signaling pathways in leukocyte-mediated inflammatory responses [56]. During asthma, Syk mobilized and recruited leukocytes, particularly eosinophils and neutrophils, from blood vessels to inflamed tissues [57]. It has been proved that Syk could bind to the immunoreceptor tyrosine-based activation motif (ITAM) in the intracellular part of immune receptors by its SH2 domain, thereby receiving and transmitting activation signals downward [53, 56].

Besides, Vav1 acts as a guanine nucleotide exchange factor and is located the downstream of T cell receptor signaling pathway, ITGAL, and Syk. It recruits leukocytes to epithelial cells and amplifies T cell development, activation, and migration, leading to an inflammatory microenvironment [58] and inside-out activates integrin to promote inflammation [59]. Mazuc et al. [60] proposed that Syk stimulated the mitogen-activated protein kinase (MAPK) signaling pathway, modulated the kinase Vav1, and generated various inflammatory mediators. The process possibly involves a crucial signaling pathway, the activation of p38 mitogen-activated protein kinase (MAPK) in asthma pathogenesis [61].

**Figure 8:**
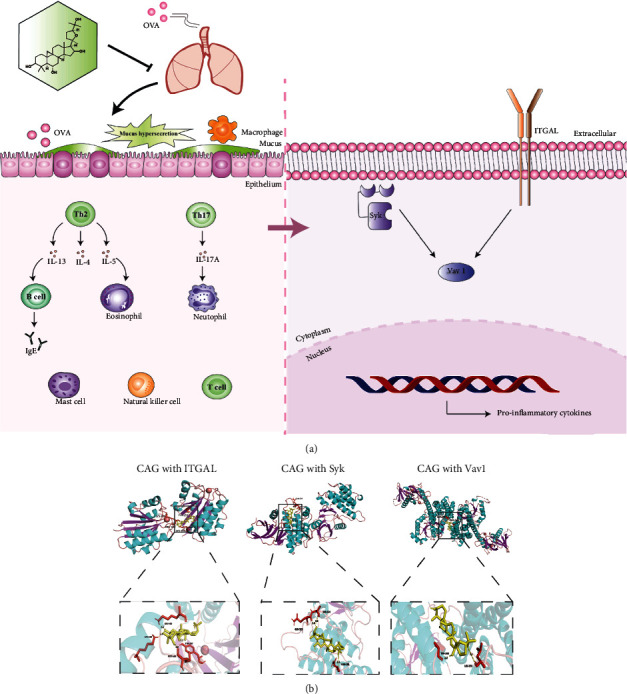
The potential mechanisms of AHR attenuation, airway inflammation relief, and mucus secretion decline in OVA-induced murine asthma model by the effects of CAG. (a) OVA induction caused immune cell infiltration that promoted Th2 cells to produce IL-4, IL-5, and IL-13. Also, Th17 cells were stimulated to generate IL-17A. Then, B cells were activated to secrete IgE by IL-13. Besides, eosinophils were recruited by IL-5 while neutrophils were boosted by IL-17A. In the course of asthma, ITGAL, Syk, and their downstream Vav1 were activated to develop the systemic immune response. They got involved in the secretion of B cells, NK cells, mast cells, and other leukocytes, leading to an inflammatory microenvironment. Of note, CAG had the potential to revert all of these processes of inflammation. (b) The predicted binding sites of CAG with ITGAL (PDB ID: 1XUO, binding energy: 8.95), Syk (PDB ID: 5Y5U, binding energy: 5.98), and Vav1 (PDB ID: 3BJI, binding energy: 5.58).

In our study, we verified that CAG dramatically repressed both the gene expression and protein expression of ITGAL, Syk, and Vav1 via qRT-PCR, western blot, and immunohistochemistry. This process was probably caused by the suppression of p38 MAPK by CAG. We suspected that CAG suppressed the upstream targets ITGAL and Syk and the downstream target Vav1 to decrease the excessive infiltration of leukocytes in the formation of asthma. Then, CAG reduced the cytokine release mediated by T cells, B cells, NK cells, and neutrophils (Figure 8(a)). The inhibition of p38 MAPK signaling by CAG might participate in this process. We also simulated the potential binding sites between CAG and ITGAL, Syk, and Vav1 to further confirm that these 3 targets remain important in the therapeutic effects of CAG in asthma (Figure 8(b)). And in future research, we will validate the core proteins ITGAL and Syk that changed the most significantly in vitro by ITGAL knockdown and Syk inhibitor to figure out whether this protein is the direct mechanism of the therapeutic effects of CAG.

## 5. Conclusion

In summary, CAG suppressed ITGAL, Syk, Vav1, and p38 MAPK expressions, contributing to the alleviation of AHR, airway inflammation, and mucus hypersecretion. Our findings proved the preventive and therapeutic effects of CAG in asthmatic mice. We also provided novel insights into the mechanisms of CAG for protecting lungs from inflammation, which suggested that the anti-inflammatory function of CAG was closely correlated with the inhibition of upstream targets ITGAL and Syk and the downstream target Vav1, probably via p38 MAPK signaling.

## Figures and Tables

**Table 1:** Primer sequences used in amplification PCR and semiquantitative RT-PCR.

Genes	Sequences	
Itgal (mouse)	Forward	ACTGACAGCCCAGGAATAGAC
	Reverse	TGAAGGACAGGATACACGGT
Syk (mouse)	Forward	5′ GAA GCC TTG CTA AGT GCG ACA 3′
	Reverse	5′ AAG TGC CGT GAA TGG GTG AC 3′
Vav1 (mouse)	Forward	5′ CAA TGA AAC CCT ACG GCA GAT 3′
	Reverse	5′ CGA CGC TCC ACT GAG GTA AT 3′
Actb (mouse)	Forward	5′ CCT CTA TGC CAA CAC AGT 3′
	Reverse	5′ AGC CAC CAA TCC ACA CAG 3′

**Table 2:** 3 DEPs regulated by CAG.

Protein ID	Gene name	Description	Peptides	Asthma model (A) vs. Normal control (N)			CAG treatment (CT) vs. Asthma model (A)		
				*P* value	Ratio	Regulated type	*P* value	Ratio	Regulated type
P24063	Itgal	Integrin alpha-L OS=Mus musculus OX=10090 GN=Itgal PE=1 SV=2	5	0.001	*1.354*	Up	0.034	**0.808**	Down
P48025	Syk	Tyrosine-protein kinase SYK OS=Mus musculus OX=10090 GN=Syk PE=1 SV=2	16	0.001	*1.487*	Up	0.041	**0.771**	Down
P27870	Vav1	Proto-oncogene vav OS=Mus musculus OX=10090 GN=Vav1 PE=1 SV=1	13	0.001	*1.430*	Up	0.036	**0.803**	Down

Italic and bold indicated upregulated or downregulated expression.

## Data Availability

The datasets used and analyzed during the study are available from the corresponding authors on reasonable request.

## Supplementary Materials

Supplementary Materials Table S1: 248 DEPs regulated by CAG. TMT-based quantitative proteomic analysis identified 248 overlapped DEPs directly regulated by CAG. Orange indicated upregulated expression, and green indicated downregulated expression (fold change > 1.2 or fold change < 0.83 and *P* value < 0.05).
