# Supplementary material for: TMT-Based Quantitative Proteomic Analysis Reveals Downregulation of ITGAL and Syk by the Effects of Cycloastragenol in OVA-Induced Asthmatic Mice
Source: Oxid Med Cell Longev. 2022 Oct 25;2022:6842530. doi: 10.1155/2022/6842530 (PMC9626231; doi:10.1155/2022/6842530)
Supplement: Supplementary Materials — Table S1: 248 DEPs regulated by CAG. TMT-based quantitative proteomic analysis identified 248 overlapped DEPs directly regulated by CAG. Orange indicated upregulated expression, and green indicated downregulated expression (fold change > 1.2 or fold change < 0.83 and P value < 0.05). [file 6842530.f1.docx]

**Table S1 248 DEPs regulated by CAG**

| 248 DEPs regulated by CAG | | | | | | | | | |
| --- | --- | --- | --- | --- | --- | --- | --- | --- | --- |
|  |  |  |  | A vs. N | | | CT vs. A | | |
| Protein ID | Gene Name | Description | Peptides | *P*-value | Ratio | Regulated Type | *P*-value | Ratio | Regulated Type |
| Q61176 | Arg1 | Arginase-1 OS=Mus musculus OX=10090 GN=Arg1 PE=1 SV=1 | 13 | 0.000423447 | 5.049638989 | up | 0.018250656 | 0.41054513 | down |
| P18581 | Slc7a2 | Cationic amino acid transporter 2 OS=Mus musculus OX=10090 GN=Slc7a2 PE=1 SV=3 | 2 | 0.000163492 | 4.004618938 | up | 0.007897355 | 0.480392157 | down |
| P11157 | Rrm2 | Ribonucleoside-diphosphate reductase subunit M2 OS=Mus musculus OX=10090 GN=Rrm2 PE=1 SV=1 | 1 | 0.001369137 | 3.477289113 | up | 0.042339204 | 0.578270786 | down |
| P02798 | Mt2 | Metallothionein-2 OS=Mus musculus OX=10090 GN=Mt2 PE=1 SV=2 | 1 | 0.002226417 | 2.880073801 | up | 0.030337588 | 0.574631646 | down |
| Q9JHW9 | Aldh1a3 | Aldehyde dehydrogenase family 1 member A3 OS=Mus musculus OX=10090 GN=Aldh1a3 PE=1 SV=1 | 7 | 0.000685535 | 2.81391201 | up | 0.012474868 | 0.546165223 | down |
| Q80YX1 | Tnc | Tenascin OS=Mus musculus OX=10090 GN=Tnc PE=1 SV=1 | 9 | 0.000764868 | 2.703151862 | up | 0.010423428 | 0.537841849 | down |
| P10923 | Spp1 | Osteopontin OS=Mus musculus OX=10090 GN=Spp1 PE=1 SV=1 | 4 | 2.69242E-05 | 2.699710145 | up | 0.042528355 | 0.561735023 | down |
| Q62266 | Sprr1a | Cornifin-A OS=Mus musculus OX=10090 GN=Sprr1a PE=1 SV=1 | 1 | 0.00256425 | 2.624415888 | up | 0.045662748 | 0.621856221 | down |
| O09046 | Il4i1 | L-amino-acid oxidase OS=Mus musculus OX=10090 GN=Il4i1 PE=1 SV=1 | 2 | 5.80681E-06 | 2.472067039 | up | 0.018581849 | 0.628926554 | down |
| Q09143 | Slc7a1 | High affinity cationic amino acid transporter 1 OS=Mus musculus OX=10090 GN=Slc7a1 PE=1 SV=1 | 1 | 4.41159E-05 | 2.434100418 | up | 0.004333174 | 0.522990976 | down |
| P01637 |  | Ig kappa chain V-V region T1 OS=Mus musculus OX=10090 PE=4 SV=1 | 2 | 0.002379208 | 2.431952663 | up | 0.01918128 | 0.579738996 | down |
| Q2VLH6 | Cd163 | Scavenger receptor cysteine-rich type 1 protein M130 OS=Mus musculus OX=10090 GN=Cd163 PE=1 SV=2 | 21 | 0.000465136 | 2.374527793 | up | 0.022624182 | 0.624318182 | down |
| P97426 | Ear1 | Eosinophil cationic protein 1 OS=Mus musculus OX=10090 GN=Ear1 PE=2 SV=1 | 3 | 0.001515407 | 2.281045752 | up | 0.013827761 | 0.545294247 | down |
| O89017 | Lgmn | Legumain OS=Mus musculus OX=10090 GN=Lgmn PE=1 SV=1 | 5 | 1.05949E-05 | 2.260042283 | up | 0.039750841 | 0.662301216 | down |
| P61514 | Rpl37a | 60S ribosomal protein L37a OS=Mus musculus OX=10090 GN=Rpl37a PE=1 SV=2 | 2 | 0.001939161 | 2.258767269 | up | 0.048228507 | 0.674429546 | down |
| P97369 | Ncf4 | Neutrophil cytosol factor 4 OS=Mus musculus OX=10090 GN=Ncf4 PE=1 SV=2 | 6 | 0.000875823 | 2.25862069 | up | 0.036140469 | 0.639139486 | down |
| Q8BVZ5 | Il33 | Interleukin-33 OS=Mus musculus OX=10090 GN=Il33 PE=1 SV=1 | 4 | 0.000451941 | 2.254201681 | up | 0.022262598 | 0.653075489 | down |
| Q9DCJ9 | Npl | N-acetylneuraminate lyase OS=Mus musculus OX=10090 GN=Npl PE=1 SV=1 | 2 | 0.000118922 | 2.232912723 | up | 0.039158455 | 0.671297386 | down |
| Q9D964 | Gatm | Glycine amidinotransferase, mitochondrial OS=Mus musculus OX=10090 GN=Gatm PE=1 SV=1 | 11 | 0.000108787 | 2.223092999 | up | 0.033324385 | 0.665569918 | down |
| P97425 | Ear2 | Eosinophil cationic protein 2 OS=Mus musculus OX=10090 GN=Ear2 PE=2 SV=1 | 4 | 0.00278673 | 2.213779129 | up | 0.036812805 | 0.60778032 | down |
| Q61830 | Mrc1 | Macrophage mannose receptor 1 OS=Mus musculus OX=10090 GN=Mrc1 PE=1 SV=2 | 40 | 0.000116911 | 2.209424084 | up | 0.022527618 | 0.680331754 | down |
| P04918 | Saa3 | Serum amyloid A-3 protein OS=Mus musculus OX=10090 GN=Saa3 PE=1 SV=1 | 2 | 9.72412E-05 | 2.131055584 | up | 0.023068056 | 0.684134961 | down |
| P16110 | Lgals3 | Galectin-3 OS=Mus musculus OX=10090 GN=Lgals3 PE=1 SV=3 | 8 | 9.36611E-05 | 2.079800499 | up | 0.026465019 | 0.677697842 | down |
| P15209 | Ntrk2 | BDNF/NT-3 growth factors receptor OS=Mus musculus OX=10090 GN=Ntrk2 PE=1 SV=1 | 1 | 0.003103885 | 2.060894386 | up | 0.007952287 | 0.592566944 | down |
| Q8JZK9 | Hmgcs1 | Hydroxymethylglutaryl-CoA synthase, cytoplasmic OS=Mus musculus OX=10090 GN=Hmgcs1 PE=1 SV=1 | 1 | 0.000728047 | 2.015461347 | up | 0.022558184 | 0.731007176 | down |
| P24452 | Capg | Macrophage-capping protein OS=Mus musculus OX=10090 GN=Capg PE=1 SV=2 | 9 | 0.000117305 | 2.005413386 | up | 0.041443152 | 0.710184049 | down |
| O70469 | Dok2 | Docking protein 2 OS=Mus musculus OX=10090 GN=Dok2 PE=1 SV=1 | 3 | 0.000184884 | 1.928402086 | up | 0.041471108 | 0.694123433 | down |
| Q62351 | Tfrc | Transferrin receptor protein 1 OS=Mus musculus OX=10090 GN=Tfrc PE=1 SV=1 | 16 | 4.1608E-05 | 1.899812734 | up | 0.010471571 | 0.691966486 | down |
| Q3TBT3 | Sting1 | Stimulator of interferon genes protein OS=Mus musculus OX=10090 GN=Sting1 PE=1 SV=2 | 4 | 0.000829989 | 1.876093966 | up | 0.027402583 | 0.676651117 | down |
| P97484 | Lilrb3 | Leukocyte immunoglobulin-like receptor subfamily B member 3 OS=Mus musculus OX=10090 GN=Lilrb3 PE=1 SV=1 | 2 | 0.000438426 | 1.855455834 | up | 0.045487295 | 0.752545825 | down |
| O08691 | Arg2 | Arginase-2, mitochondrial OS=Mus musculus OX=10090 GN=Arg2 PE=1 SV=1 | 1 | 0.000212829 | 1.85499515 | up | 0.03715949 | 0.813856209 | down |
| Q09014 | Ncf1 | Neutrophil cytosol factor 1 OS=Mus musculus OX=10090 GN=Ncf1 PE=1 SV=3 | 14 | 0.001694865 | 1.851141553 | up | 0.034851646 | 0.680315738 | down |
| Q9QZL0 | Ripk3 | Receptor-interacting serine/threonine-protein kinase 3 OS=Mus musculus OX=10090 GN=Ripk3 PE=1 SV=2 | 5 | 1.72608E-05 | 1.842883549 | up | 0.021220292 | 0.714142427 | down |
| O70401 | Tspan6 | Tetraspanin-6 OS=Mus musculus OX=10090 GN=Tspan6 PE=1 SV=1 | 1 | 0.013613972 | 1.780309936 | up | 0.046935086 | 0.7421915 | down |
| Q9CQI6 | Cotl1 | Coactosin-like protein OS=Mus musculus OX=10090 GN=Cotl1 PE=1 SV=3 | 12 | 2.19407E-05 | 1.767012168 | up | 0.022447744 | 0.729150727 | down |
| P30204 | Msr1 | Macrophage scavenger receptor types I and II OS=Mus musculus OX=10090 GN=Msr1 PE=1 SV=3 | 1 | 0.000269528 | 1.764887517 | up | 0.038747933 | 0.683329168 | down |
| O88379 | Baz1a | Bromodomain adjacent to zinc finger domain protein 1A OS=Mus musculus OX=10090 GN=Baz1a PE=1 SV=3 | 4 | 0.001441763 | 1.755839577 | up | 0.018992242 | 0.689508032 | down |
| P10605 | Ctsb | Cathepsin B OS=Mus musculus OX=10090 GN=Ctsb PE=1 SV=2 | 11 | 0.000113507 | 1.745762712 | up | 0.0293784 | 0.726622381 | down |
| Q61233 | Lcp1 | Plastin-2 OS=Mus musculus OX=10090 GN=Lcp1 PE=1 SV=4 | 36 | 9.03474E-05 | 1.745133545 | up | 0.031719791 | 0.761348898 | down |
| Q69ZK0 | Prex1 | Phosphatidylinositol 3,4,5-trisphosphate-dependent Rac exchanger 1 protein OS=Mus musculus OX=10090 GN=Prex1 PE=1 SV=2 | 2 | 0.001186393 | 1.711719418 | up | 0.003318235 | 0.664417791 | down |
| Q61333 | Tnfaip2 | Tumor necrosis factor alpha-induced protein 2 OS=Mus musculus OX=10090 GN=Tnfaip2 PE=1 SV=2 | 1 | 0.002146079 | 1.70247191 | up | 0.043083118 | 0.788278775 | down |
| P12032 | Timp1 | Metalloproteinase inhibitor 1 OS=Mus musculus OX=10090 GN=Timp1 PE=1 SV=2 | 2 | 0.03265366 | 1.67110573 | up | 0.022853806 | 0.574981888 | down |
| Q640N3 | Arhgap30 | Rho GTPase-activating protein 30 OS=Mus musculus OX=10090 GN=Arhgap30 PE=1 SV=3 | 2 | 0.000784968 | 1.661465106 | up | 0.023619011 | 0.745630055 | down |
| Q80YW0 | Cyth4 | Cytohesin-4 OS=Mus musculus OX=10090 GN=Cyth4 PE=1 SV=1 | 2 | 0.001926635 | 1.658924731 | up | 0.028230518 | 0.730878921 | down |
| P18155 | Mthfd2 | Bifunctional methylenetetrahydrofolate dehydrogenase/cyclohydrolase, mitochondrial OS=Mus musculus OX=10090 GN=Mthfd2 PE=1 SV=1 | 1 | 8.60501E-05 | 1.657871972 | up | 0.002619781 | 0.744586486 | down |
| Q3T9E4 | Tgtp2 | T-cell-specific guanine nucleotide triphosphate-binding protein 2 OS=Mus musculus OX=10090 GN=Tgtp2 PE=1 SV=2 | 6 | 0.008567908 | 1.643894108 | up | 0.027310432 | 0.729350649 | down |
| Q8BJW5 | Nol11 | Nucleolar protein 11 OS=Mus musculus OX=10090 GN=Nol11 PE=2 SV=1 | 1 | 0.007903164 | 1.619250426 | up | 0.030656044 | 0.749079432 | down |
| O89053 | Coro1a | Coronin-1A OS=Mus musculus OX=10090 GN=Coro1a PE=1 SV=5 | 21 | 0.000849406 | 1.61085595 | up | 0.021772849 | 0.712026957 | down |
| Q9D8Y7 | Tnfaip8l2 | Tumor necrosis factor alpha-induced protein 8-like protein 2 OS=Mus musculus OX=10090 GN=Tnfaip8l2 PE=1 SV=1 | 2 | 0.000925944 | 1.609234716 | up | 0.012036823 | 0.769659936 | down |
| Q8CG47 | Smc4 | Structural maintenance of chromosomes protein 4 OS=Mus musculus OX=10090 GN=Smc4 PE=1 SV=1 | 8 | 0.000222985 | 1.60910251 | up | 0.02715273 | 0.757599789 | down |
| Q80SU7 | Gvin1 | Interferon-induced very large GTPase 1 OS=Mus musculus OX=10090 GN=Gvin1 PE=1 SV=1 | 1 | 0.001797411 | 1.607235142 | up | 0.03834195 | 0.789121115 | down |
| Q7TMF2 | Eri1 | 3'-5' exoribonuclease 1 OS=Mus musculus OX=10090 GN=Eri1 PE=1 SV=2 | 2 | 0.000837277 | 1.601440068 | up | 0.019119636 | 0.755620206 | down |
| Q8K1X4 | Nckap1l | Nck-associated protein 1-like OS=Mus musculus OX=10090 GN=Nckap1l PE=1 SV=1 | 10 | 0.000209512 | 1.598720682 | up | 0.031418446 | 0.775140037 | down |
| Q9D142 | Nudt14 | Uridine diphosphate glucose pyrophosphatase NUDT14 OS=Mus musculus OX=10090 GN=Nudt14 PE=1 SV=1 | 1 | 0.001613378 | 1.597844828 | up | 0.042609229 | 0.801996223 | down |
| Q61599 | Arhgdib | Rho GDP-dissociation inhibitor 2 OS=Mus musculus OX=10090 GN=Arhgdib PE=1 SV=3 | 10 | 0.000931436 | 1.593283582 | up | 0.030919136 | 0.714025501 | down |
| Q61462 | Cyba | Cytochrome b-245 light chain OS=Mus musculus OX=10090 GN=Cyba PE=1 SV=3 | 3 | 0.001656832 | 1.589323245 | up | 0.041739715 | 0.751653002 | down |
| Q8R4K2 | Irak4 | Interleukin-1 receptor-associated kinase 4 OS=Mus musculus OX=10090 GN=Irak4 PE=1 SV=1 | 2 | 0.000304337 | 1.588210167 | up | 0.040471831 | 0.791016676 | down |
| P17918 | Pcna | Proliferating cell nuclear antigen OS=Mus musculus OX=10090 GN=Pcna PE=1 SV=2 | 8 | 4.522E-05 | 1.582532386 | up | 0.030971053 | 0.745180882 | down |
| Q9D1R9 | Rpl34 | 60S ribosomal protein L34 OS=Mus musculus OX=10090 GN=Rpl34 PE=1 SV=2 | 5 | 0.002289512 | 1.581663113 | up | 0.038008304 | 0.794284174 | down |
| P29351 | Ptpn6 | Tyrosine-protein phosphatase non-receptor type 6 OS=Mus musculus OX=10090 GN=Ptpn6 PE=1 SV=2 | 24 | 0.001147764 | 1.576391796 | up | 0.030061398 | 0.755443441 | down |
| O70145 | Ncf2 | Neutrophil cytosol factor 2 OS=Mus musculus OX=10090 GN=Ncf2 PE=1 SV=1 | 12 | 0.001441628 | 1.576086957 | up | 0.041377669 | 0.752519894 | down |
| Q9CPR4 | Rpl17 | 60S ribosomal protein L17 OS=Mus musculus OX=10090 GN=Rpl17 PE=1 SV=3 | 5 | 0.000102501 | 1.574658703 | up | 0.032758269 | 0.803305337 | down |
| Q6ZWY8 | Tmsb10 | Thymosin beta-10 OS=Mus musculus OX=10090 GN=Tmsb10 PE=1 SV=3 | 4 | 0.000641423 | 1.562244062 | up | 0.03457406 | 0.719003932 | down |
| P06800 | Ptprc | Receptor-type tyrosine-protein phosphatase C OS=Mus musculus OX=10090 GN=Ptprc PE=1 SV=4 | 26 | 0.000763835 | 1.561506276 | up | 0.032604754 | 0.771436227 | down |
| Q9R060 | Nubp1 | Cytosolic Fe-S cluster assembly factor NUBP1 OS=Mus musculus OX=10090 GN=Nubp1 PE=1 SV=1 | 2 | 0.000243189 | 1.559780313 | up | 0.029663126 | 0.796587216 | down |
| Q9JJ94 | Ssna1 | Sjoegren syndrome nuclear autoantigen 1 homolog OS=Mus musculus OX=10090 GN=Ssna1 PE=1 SV=1 | 1 | 8.90507E-05 | 1.558614935 | up | 0.011266672 | 0.767398287 | down |
| Q61881 | Mcm7 | DNA replication licensing factor MCM7 OS=Mus musculus OX=10090 GN=Mcm7 PE=1 SV=1 | 10 | 4.74126E-05 | 1.550755034 | up | 0.040718774 | 0.789288612 | down |
| O09159 | Man2b1 | Lysosomal alpha-mannosidase OS=Mus musculus OX=10090 GN=Man2b1 PE=1 SV=4 | 9 | 0.000849122 | 1.544282744 | up | 0.04903886 | 0.775713516 | down |
| Q99JH1 | Rpp25l | Ribonuclease P protein subunit p25-like protein OS=Mus musculus OX=10090 GN=Rpp25l PE=1 SV=1 | 1 | 0.00351297 | 1.539101498 | up | 0.013965034 | 0.782702703 | down |
| Q99JF5 | Mvd | Diphosphomevalonate decarboxylase OS=Mus musculus OX=10090 GN=Mvd PE=1 SV=2 | 4 | 8.15433E-05 | 1.537200504 | up | 0.030185412 | 0.810773858 | down |
| Q3UMW7 | Mapkapk3 | MAP kinase-activated protein kinase 3 OS=Mus musculus OX=10090 GN=Mapkapk3 PE=1 SV=2 | 5 | 0.000554084 | 1.533866218 | up | 0.03123586 | 0.816236972 | down |
| P70248 | Myo1f | Unconventional myosin-If OS=Mus musculus OX=10090 GN=Myo1f PE=1 SV=1 | 14 | 0.001133251 | 1.533498759 | up | 0.042579981 | 0.775080906 | down |
| P63028 | Tpt1 | Translationally-controlled tumor protein OS=Mus musculus OX=10090 GN=Tpt1 PE=1 SV=1 | 9 | 0.000118453 | 1.533057851 | up | 0.01281557 | 0.773584906 | down |
| O35375 | Nrp2 | Neuropilin-2 OS=Mus musculus OX=10090 GN=Nrp2 PE=1 SV=2 | 2 | 0.000132795 | 1.528649101 | up | 0.0422229 | 0.807934337 | down |
| Q8BLR5 | Psd4 | PH and SEC7 domain-containing protein 4 OS=Mus musculus OX=10090 GN=Psd4 PE=1 SV=1 | 1 | 0.016760323 | 1.528031809 | up | 0.009138892 | 0.687483737 | down |
| Q5XG71 | Utp20 | Small subunit processome component 20 homolog OS=Mus musculus OX=10090 GN=Utp20 PE=1 SV=2 | 2 | 0.0051422 | 1.524123711 | up | 0.023653289 | 0.778679654 | down |
| P28867 | Prkcd | Protein kinase C delta type OS=Mus musculus OX=10090 GN=Prkcd PE=1 SV=3 | 21 | 7.42827E-05 | 1.515138946 | up | 0.038119033 | 0.803722967 | down |
| Q91WP6 | Serpina3n | Serine protease inhibitor A3N OS=Mus musculus OX=10090 GN=Serpina3n PE=1 SV=1 | 16 | 0.02049175 | 1.50999592 | up | 0.03466964 | 0.769251554 | down |
| Q8K0C4 | Cyp51a1 | Lanosterol 14-alpha demethylase OS=Mus musculus OX=10090 GN=Cyp51a1 PE=1 SV=1 | 6 | 0.001890457 | 1.504016064 | up | 0.016024695 | 0.738317757 | down |
| P49718 | Mcm5 | DNA replication licensing factor MCM5 OS=Mus musculus OX=10090 GN=Mcm5 PE=1 SV=2 | 9 | 0.000172993 | 1.503822938 | up | 0.022071218 | 0.743644635 | down |
| P08030 | Aprt | Adenine phosphoribosyltransferase OS=Mus musculus OX=10090 GN=Aprt PE=1 SV=2 | 4 | 0.001176152 | 1.502079867 | up | 0.047778763 | 0.826917751 | down |
| Q8C3J5 | Dock2 | Dedicator of cytokinesis protein 2 OS=Mus musculus OX=10090 GN=Dock2 PE=1 SV=3 | 22 | 0.001413033 | 1.501427989 | up | 0.042891705 | 0.779891304 | down |
| Q9JM51 | Ptges | Prostaglandin E synthase OS=Mus musculus OX=10090 GN=Ptges PE=1 SV=1 | 2 | 0.000682445 | 1.49752883 | up | 0.032764991 | 0.807480748 | down |
| Q9D7A6 | Srp19 | Signal recognition particle 19 kDa protein OS=Mus musculus OX=10090 GN=Srp19 PE=1 SV=1 | 2 | 0.001990193 | 1.49626556 | up | 0.043733545 | 0.827509706 | down |
| Q99M31 | Hspa14 | Heat shock 70 kDa protein 14 OS=Mus musculus OX=10090 GN=Hspa14 PE=1 SV=2 | 6 | 0.001849191 | 1.492263844 | up | 0.023649749 | 0.784993179 | down |
| Q61263 | Soat1 | Sterol O-acyltransferase 1 OS=Mus musculus OX=10090 GN=Soat1 PE=1 SV=2 | 4 | 0.000680512 | 1.491918773 | up | 0.017656907 | 0.83 | down |
| Q6ZWV7 | Rpl35 | 60S ribosomal protein L35 OS=Mus musculus OX=10090 GN=Rpl35 PE=1 SV=1 | 2 | 0.000100998 | 1.491372227 | up | 0.028866972 | 0.809090909 | down |
| O70251 | Eef1b | Elongation factor 1-beta OS=Mus musculus OX=10090 GN=Eef1b PE=1 SV=5 | 7 | 0.00022315 | 1.490115321 | up | 0.036553759 | 0.81647319 | down |
| P48025 | Syk | Tyrosine-protein kinase SYK OS=Mus musculus OX=10090 GN=Syk PE=1 SV=2 | 16 | 0.001306162 | 1.486879289 | up | 0.040612068 | 0.77083899 | down |
| P49710 | Hcls1 | Hematopoietic lineage cell-specific protein OS=Mus musculus OX=10090 GN=Hcls1 PE=1 SV=2 | 18 | 0.000199744 | 1.486596263 | up | 0.02081399 | 0.786338798 | down |
| P61358 | Rpl27 | 60S ribosomal protein L27 OS=Mus musculus OX=10090 GN=Rpl27 PE=1 SV=2 | 4 | 0.000127866 | 1.484897959 | up | 0.015684582 | 0.800439802 | down |
| P43404 | Zap70 | Tyrosine-protein kinase ZAP-70 OS=Mus musculus OX=10090 GN=Zap70 PE=1 SV=3 | 2 | 0.020414923 | 1.477932406 | up | 0.007615884 | 0.745224644 | down |
| P83882 | Rpl36a | 60S ribosomal protein L36a OS=Mus musculus OX=10090 GN=Rpl36a PE=3 SV=2 | 7 | 0.000102251 | 1.475819672 | up | 0.028599632 | 0.82171619 | down |
| P62274 | Rps29 | 40S ribosomal protein S29 OS=Mus musculus OX=10090 GN=Rps29 PE=1 SV=2 | 2 | 0.000498675 | 1.473939394 | up | 0.025972853 | 0.788925439 | down |
| P62830 | Rpl23 | 60S ribosomal protein L23 OS=Mus musculus OX=10090 GN=Rpl23 PE=1 SV=1 | 8 | 0.000647271 | 1.473813421 | up | 0.023184719 | 0.820377568 | down |
| Q8CD92 | Ttc27 | Tetratricopeptide repeat protein 27 OS=Mus musculus OX=10090 GN=Ttc27 PE=1 SV=2 | 2 | 0.002447302 | 1.469817315 | up | 0.011315094 | 0.751688733 | down |
| P58252 | Eef2 | Elongation factor 2 OS=Mus musculus OX=10090 GN=Eef2 PE=1 SV=2 | 45 | 0.000185872 | 1.458080194 | up | 0.033273692 | 0.814444444 | down |
| Q6A051 | Atrnl1 | Attractin-like protein 1 OS=Mus musculus OX=10090 GN=Atrnl1 PE=1 SV=2 | 2 | 0.003168947 | 1.457779515 | up | 0.043382719 | 0.727540896 | down |
| Q9JLF6 | Tgm1 | Protein-glutamine gamma-glutamyltransferase K OS=Mus musculus OX=10090 GN=Tgm1 PE=1 SV=2 | 4 | 0.000247579 | 1.455578093 | up | 0.042765576 | 0.821348941 | down |
| P68040 | Rack1 | Receptor of activated protein C kinase 1 OS=Mus musculus OX=10090 GN=Rack1 PE=1 SV=3 | 17 | 6.36076E-05 | 1.455281263 | up | 0.029452096 | 0.815628476 | down |
| Q8BG48 | Stk17b | Serine/threonine-protein kinase 17B OS=Mus musculus OX=10090 GN=Stk17b PE=1 SV=1 | 2 | 0.00284135 | 1.453474676 | up | 0.009858116 | 0.743111831 | down |
| P15307 | Rel | Proto-oncogene c-Rel OS=Mus musculus OX=10090 GN=Rel PE=1 SV=2 | 6 | 0.000469091 | 1.452694611 | up | 0.019950474 | 0.784830998 | down |
| Q99N95 | Mrpl3 | 39S ribosomal protein L3, mitochondrial OS=Mus musculus OX=10090 GN=Mrpl3 PE=1 SV=1 | 1 | 0.003085983 | 1.45254902 | up | 0.028904205 | 0.74163067 | down |
| P21981 | Tgm2 | Protein-glutamine gamma-glutamyltransferase 2 OS=Mus musculus OX=10090 GN=Tgm2 PE=1 SV=4 | 29 | 0.004095026 | 1.451369591 | up | 0.011491133 | 0.772702407 | down |
| P70429 | Evl | Ena/VASP-like protein OS=Mus musculus OX=10090 GN=Evl PE=1 SV=2 | 14 | 0.001900635 | 1.449637389 | up | 0.047291075 | 0.811839911 | down |
| P62270 | Rps18 | 40S ribosomal protein S18 OS=Mus musculus OX=10090 GN=Rps18 PE=1 SV=3 | 7 | 1.16144E-05 | 1.446978792 | up | 0.026958392 | 0.798119469 | down |
| O55142 | Rpl35a | 60S ribosomal protein L35a OS=Mus musculus OX=10090 GN=Rpl35a PE=1 SV=2 | 3 | 0.002415495 | 1.446240905 | up | 0.038499716 | 0.823644494 | down |
| P62242 | Rps8 | 40S ribosomal protein S8 OS=Mus musculus OX=10090 GN=Rps8 PE=1 SV=2 | 10 | 3.99487E-05 | 1.446110211 | up | 0.046582209 | 0.829924349 | down |
| O88796 | Rpp30 | Ribonuclease P protein subunit p30 OS=Mus musculus OX=10090 GN=Rpp30 PE=1 SV=1 | 1 | 0.016031105 | 1.439744613 | up | 0.041399583 | 0.799611973 | down |
| P62281 | Rps11 | 40S ribosomal protein S11 OS=Mus musculus OX=10090 GN=Rps11 PE=1 SV=3 | 9 | 0.000486785 | 1.438985099 | up | 0.042477675 | 0.823957459 | down |
| Q9DBE9 | Ftsj3 | pre-rRNA 2'-O-ribose RNA methyltransferase FTSJ3 OS=Mus musculus OX=10090 GN=Ftsj3 PE=1 SV=1 | 3 | 3.89939E-05 | 1.434367542 | up | 0.015155503 | 0.79839157 | down |
| A2BE28 | Las1l | Ribosomal biogenesis protein LAS1L OS=Mus musculus OX=10090 GN=Las1l PE=1 SV=1 | 3 | 0.005616371 | 1.4328 | up | 0.024447129 | 0.814628699 | down |
| Q9JLM8 | Dclk1 | Serine/threonine-protein kinase DCLK1 OS=Mus musculus OX=10090 GN=Dclk1 PE=1 SV=1 | 4 | 0.001957995 | 1.432769726 | up | 0.006786667 | 0.830851363 | down |
| Q7TMR0 | Prcp | Lysosomal Pro-X carboxypeptidase OS=Mus musculus OX=10090 GN=Prcp PE=1 SV=2 | 3 | 0.002919769 | 1.430455635 | up | 0.025896456 | 0.815870355 | down |
| P27870 | Vav1 | Proto-oncogene vav OS=Mus musculus OX=10090 GN=Vav1 PE=1 SV=1 | 13 | 0.001293886 | 1.429650238 | up | 0.035773517 | 0.802613289 | down |
| O70456 | Sfn | 14-3-3 protein sigma OS=Mus musculus OX=10090 GN=Sfn PE=1 SV=2 | 9 | 0.049708613 | 1.419984076 | up | 0.02732681 | 0.819175778 | down |
| Q6ZWU9 | Rps27 | 40S ribosomal protein S27 OS=Mus musculus OX=10090 GN=Rps27 PE=1 SV=3 | 3 | 0.001821809 | 1.419123506 | up | 0.049857701 | 0.822290848 | down |
| P42225 | Stat1 | Signal transducer and activator of transcription 1 OS=Mus musculus OX=10090 GN=Stat1 PE=1 SV=1 | 19 | 0.00366789 | 1.417756639 | up | 0.019349281 | 0.810455689 | down |
| P62900 | Rpl31 | 60S ribosomal protein L31 OS=Mus musculus OX=10090 GN=Rpl31 PE=1 SV=1 | 7 | 9.23345E-05 | 1.416201117 | up | 0.037388393 | 0.828965906 | down |
| P47963 | Rpl13 | 60S ribosomal protein L13 OS=Mus musculus OX=10090 GN=Rpl13 PE=1 SV=3 | 12 | 5.18388E-05 | 1.413492063 | up | 0.039731425 | 0.819483436 | down |
| Q9ERL7 | Gmfg | Glia maturation factor gamma OS=Mus musculus OX=10090 GN=Gmfg PE=1 SV=1 | 3 | 0.001699118 | 1.413265306 | up | 0.0196055 | 0.791724521 | down |
| Q8BGA5 | Krr1 | KRR1 small subunit processome component homolog OS=Mus musculus OX=10090 GN=Krr1 PE=2 SV=1 | 1 | 0.02023504 | 1.412933385 | up | 0.003366588 | 0.773642128 | down |
| P14131 | Rps16 | 40S ribosomal protein S16 OS=Mus musculus OX=10090 GN=Rps16 PE=1 SV=4 | 8 | 0.000429426 | 1.41183479 | up | 0.034283481 | 0.823347398 | down |
| P25444 | Rps2 | 40S ribosomal protein S2 OS=Mus musculus OX=10090 GN=Rps2 PE=1 SV=3 | 10 | 0.000718002 | 1.406237663 | up | 0.029054966 | 0.815272319 | down |
| P30681 | Hmgb2 | High mobility group protein B2 OS=Mus musculus OX=10090 GN=Hmgb2 PE=1 SV=3 | 22 | 0.001235152 | 1.39497448 | up | 0.041475492 | 0.816211652 | down |
| Q9Z110 | Aldh18a1 | Delta-1-pyrroline-5-carboxylate synthase OS=Mus musculus OX=10090 GN=Aldh18a1 PE=1 SV=2 | 7 | 5.86362E-06 | 1.391252955 | up | 0.031572596 | 0.830359671 | down |
| Q921Z5 | Tnfaip8 | Tumor necrosis factor alpha-induced protein 8 OS=Mus musculus OX=10090 GN=Tnfaip8 PE=1 SV=1 | 4 | 0.000100905 | 1.388586957 | up | 0.024932074 | 0.795638803 | down |
| Q91WM3 | Rrp9 | U3 small nucleolar RNA-interacting protein 2 OS=Mus musculus OX=10090 GN=Rrp9 PE=1 SV=1 | 2 | 0.009812963 | 1.388413686 | up | 0.023901152 | 0.799775973 | down |
| P62702 | Rps4x | 40S ribosomal protein S4, X isoform OS=Mus musculus OX=10090 GN=Rps4x PE=1 SV=2 | 14 | 0.000202883 | 1.388147567 | up | 0.028256945 | 0.82386203 | down |
| P97770 | Thumpd3 | THUMP domain-containing protein 3 OS=Mus musculus OX=10090 GN=Thumpd3 PE=1 SV=1 | 3 | 0.006234 | 1.386970173 | up | 0.031614512 | 0.8254103 | down |
| Q8C3X4 | Guf1 | Translation factor Guf1, mitochondrial OS=Mus musculus OX=10090 GN=Guf1 PE=1 SV=1 | 1 | 0.000138592 | 1.382872504 | up | 0.015816612 | 0.776450986 | down |
| Q8R5A3 | Apbb1ip | Amyloid beta A4 precursor protein-binding family B member 1-interacting protein OS=Mus musculus OX=10090 GN=Apbb1ip PE=1 SV=2 | 12 | 7.67178E-05 | 1.375632542 | up | 0.003809893 | 0.819185059 | down |
| Q99J87 | Dhx58 | Probable ATP-dependent RNA helicase DHX58 OS=Mus musculus OX=10090 GN=Dhx58 PE=1 SV=2 | 3 | 0.001434211 | 1.370455415 | up | 0.035930688 | 0.783859257 | down |
| Q7TPV4 | Mybbp1a | Myb-binding protein 1A OS=Mus musculus OX=10090 GN=Mybbp1a PE=1 SV=2 | 22 | 0.000143931 | 1.370327103 | up | 0.012619791 | 0.827507815 | down |
| Q60787 | Lcp2 | Lymphocyte cytosolic protein 2 OS=Mus musculus OX=10090 GN=Lcp2 PE=1 SV=2 | 2 | 0.000175787 | 1.364057077 | up | 0.028309341 | 0.81198756 | down |
| Q3UND0 | Skap2 | Src kinase-associated phosphoprotein 2 OS=Mus musculus OX=10090 GN=Skap2 PE=1 SV=2 | 1 | 0.006178247 | 1.362670713 | up | 0.024739916 | 0.771993318 | down |
| Q80W93 | Hydin | Hydrocephalus-inducing protein OS=Mus musculus OX=10090 GN=Hydin PE=1 SV=2 | 1 | 0.000343646 | 1.361003861 | up | 0.00531445 | 0.818723404 | down |
| Q8BYW1 | Arhgap25 | Rho GTPase-activating protein 25 OS=Mus musculus OX=10090 GN=Arhgap25 PE=1 SV=2 | 14 | 0.000814263 | 1.357252494 | up | 0.048491148 | 0.807463953 | down |
| P24063 | Itgal | Integrin alpha-L OS=Mus musculus OX=10090 GN=Itgal PE=1 SV=2 | 5 | 0.001448271 | 1.354022989 | up | 0.033790851 | 0.80786644 | down |
| P17182 | Eno1 | Alpha-enolase OS=Mus musculus OX=10090 GN=Eno1 PE=1 SV=3 | 26 | 4.59849E-05 | 1.353302611 | up | 0.016280672 | 0.815266742 | down |
| E9Q634 | Myo1e | Unconventional myosin-Ie OS=Mus musculus OX=10090 GN=Myo1e PE=1 SV=1 | 15 | 0.000511867 | 1.34305449 | up | 0.010898617 | 0.826857143 | down |
| P62082 | Rps7 | 40S ribosomal protein S7 OS=Mus musculus OX=10090 GN=Rps7 PE=2 SV=1 | 10 | 0.000360394 | 1.3406298 | up | 0.017144148 | 0.831853337 | down |
| Q9JIK5 | Ddx21 | Nucleolar RNA helicase 2 OS=Mus musculus OX=10090 GN=Ddx21 PE=1 SV=3 | 21 | 0.000353362 | 1.338924895 | up | 0.011532886 | 0.815774487 | down |
| P97814 | Pstpip1 | Proline-serine-threonine phosphatase-interacting protein 1 OS=Mus musculus OX=10090 GN=Pstpip1 PE=1 SV=1 | 2 | 0.014342551 | 1.334703026 | up | 0.016627352 | 0.769661349 | down |
| P45700 | Man1a1 | Mannosyl-oligosaccharide 1,2-alpha-mannosidase IA OS=Mus musculus OX=10090 GN=Man1a1 PE=1 SV=1 | 3 | 0.001808697 | 1.333079848 | up | 0.043020606 | 0.817170565 | down |
| Q8R550 | Sh3kbp1 | SH3 domain-containing kinase-binding protein 1 OS=Mus musculus OX=10090 GN=Sh3kbp1 PE=1 SV=1 | 13 | 0.000442016 | 1.332954115 | up | 0.04395551 | 0.810241821 | down |
| Q9EQ61 | Pes1 | Pescadillo homolog OS=Mus musculus OX=10090 GN=Pes1 PE=1 SV=1 | 6 | 4.98594E-05 | 1.329666161 | up | 0.002730531 | 0.815691869 | down |
| P60843 | Eif4a1 | Eukaryotic initiation factor 4A-I OS=Mus musculus OX=10090 GN=Eif4a1 PE=1 SV=1 | 18 | 1.26823E-05 | 1.327238095 | up | 0.013344735 | 0.829793341 | down |
| Q3V3R1 | Mthfd1l | Monofunctional C1-tetrahydrofolate synthase, mitochondrial OS=Mus musculus OX=10090 GN=Mthfd1l PE=1 SV=2 | 13 | 0.003807529 | 1.326623623 | up | 0.040449235 | 0.823074721 | down |
| Q9Z2X2 | Psmd10 | 26S proteasome non-ATPase regulatory subunit 10 OS=Mus musculus OX=10090 GN=Psmd10 PE=1 SV=3 | 4 | 0.000320181 | 1.324857685 | up | 0.022668607 | 0.823259811 | down |
| Q61152 | Ptpn18 | Tyrosine-protein phosphatase non-receptor type 18 OS=Mus musculus OX=10090 GN=Ptpn18 PE=1 SV=1 | 1 | 0.005144626 | 1.317081979 | up | 0.008903515 | 0.708253359 | down |
| Q8BY71 | Hat1 | Histone acetyltransferase type B catalytic subunit OS=Mus musculus OX=10090 GN=Hat1 PE=1 SV=1 | 5 | 0.010591911 | 1.312127976 | up | 0.02342598 | 0.790189963 | down |
| P15702 | Spn | Leukosialin OS=Mus musculus OX=10090 GN=Spn PE=1 SV=1 | 2 | 0.014484011 | 1.299253731 | up | 0.043416053 | 0.814761631 | down |
| Q8R3K3 | Ptcd2 | Pentatricopeptide repeat-containing protein 2, mitochondrial OS=Mus musculus OX=10090 GN=Ptcd2 PE=1 SV=1 | 1 | 0.003636628 | 1.297236744 | up | 0.035181272 | 0.81980426 | down |
| P22682 | Cbl | E3 ubiquitin-protein ligase CBL OS=Mus musculus OX=10090 GN=Cbl PE=1 SV=3 | 5 | 0.005136474 | 1.296857671 | up | 0.016092007 | 0.794754846 | down |
| Q80UZ2 | Sdad1 | Protein SDA1 homolog OS=Mus musculus OX=10090 GN=Sdad1 PE=1 SV=1 | 1 | 0.023467013 | 1.289021658 | up | 0.046919265 | 0.831691773 | down |
| Q6PD19 | Armh3 | Armadillo-like helical domain-containing protein 3 OS=Mus musculus OX=10090 GN=Armh3 PE=1 SV=2 | 2 | 0.004879555 | 1.284069811 | up | 0.012403511 | 0.82388664 | down |
| Q8BH65 | Dennd6a | Protein DENND6A OS=Mus musculus OX=10090 GN=Dennd6a PE=1 SV=1 | 2 | 0.031677742 | 1.272131148 | up | 0.046863718 | 0.791237113 | down |
| Q3TBD2 | Arhgap45 | Rho GTPase-activating protein 45 OS=Mus musculus OX=10090 GN=Arhgap45 PE=1 SV=2 | 14 | 0.001221328 | 1.269911504 | up | 0.009212872 | 0.825203252 | down |
| Q62179 | Sema4b | Semaphorin-4B OS=Mus musculus OX=10090 GN=Sema4b PE=1 SV=2 | 3 | 0.000206395 | 1.26900369 | up | 0.015118029 | 0.829020064 | down |
| P42230 | Stat5a | Signal transducer and activator of transcription 5A OS=Mus musculus OX=10090 GN=Stat5a PE=1 SV=1 | 11 | 0.011651653 | 1.268468468 | up | 0.00824882 | 0.76875 | down |
| Q9ET22 | Dpp7 | Dipeptidyl peptidase 2 OS=Mus musculus OX=10090 GN=Dpp7 PE=1 SV=2 | 2 | 0.014765357 | 1.236422882 | up | 0.039491755 | 0.826939971 | down |
| Q61576 | Fkbp10 | Peptidyl-prolyl cis-trans isomerase FKBP10 OS=Mus musculus OX=10090 GN=Fkbp10 PE=1 SV=2 | 8 | 0.011409156 | 1.219320215 | up | 0.021650353 | 0.820715962 | down |
| O08528 | Hk2 | Hexokinase-2 OS=Mus musculus OX=10090 GN=Hk2 PE=1 SV=1 | 18 | 0.043895514 | 1.214183892 | up | 0.047913386 | 0.817728207 | down |
| Q9JME5 | Ap3b2 | AP-3 complex subunit beta-2 OS=Mus musculus OX=10090 GN=Ap3b2 PE=1 SV=2 | 3 | 0.000774613 | 0.47607116 | down | 0.043017553 | 1.636315789 | up |
| P10922 | H1-0 | Histone H1.0 OS=Mus musculus OX=10090 GN=H1-0 PE=2 SV=4 | 7 | 2.88456E-06 | 0.557477382 | down | 0.027392409 | 1.502625298 | up |
| P33622 | Apoc3 | Apolipoprotein C-III OS=Mus musculus OX=10090 GN=Apoc3 PE=1 SV=2 | 4 | 0.035589626 | 0.557746479 | down | 0.044251217 | 1.338842975 | up |
| Q9WTR5 | Cdh13 | Cadherin-13 OS=Mus musculus OX=10090 GN=Cdh13 PE=1 SV=2 | 7 | 0.003466889 | 0.583023873 | down | 0.021814773 | 1.37989081 | up |
| Q8VEM1 | Rnf130 | E3 ubiquitin-protein ligase RNF130 OS=Mus musculus OX=10090 GN=Rnf130 PE=2 SV=1 | 2 | 0.009348559 | 0.587086696 | down | 0.040809973 | 1.288248337 | up |
| Q62000 | Ogn | Mimecan OS=Mus musculus OX=10090 GN=Ogn PE=1 SV=1 | 7 | 0.01068453 | 0.58843808 | down | 0.045604181 | 1.356016224 | up |
| Q80WR1 | Tspan18 | Tetraspanin-18 OS=Mus musculus OX=10090 GN=Tspan18 PE=2 SV=1 | 3 | 0.00064737 | 0.589334799 | down | 0.045564665 | 1.501865672 | up |
| O08532 | Cacna2d1 | Voltage-dependent calcium channel subunit alpha-2/delta-1 OS=Mus musculus OX=10090 GN=Cacna2d1 PE=1 SV=1 | 9 | 0.000264629 | 0.591519621 | down | 0.04684701 | 1.316562778 | up |
| P52927 | Hmga2 | High mobility group protein HMGI-C OS=Mus musculus OX=10090 GN=Hmga2 PE=1 SV=1 | 3 | 0.005280107 | 0.621359223 | down | 0.037474429 | 1.408482143 | up |
| P70257 | Nfix | Nuclear factor 1 X-type OS=Mus musculus OX=10090 GN=Nfix PE=1 SV=2 | 4 | 8.5417E-05 | 0.621841891 | down | 0.025430947 | 1.322848405 | up |
| O70423 | Aoc3 | Membrane primary amine oxidase OS=Mus musculus OX=10090 GN=Aoc3 PE=1 SV=3 | 6 | 0.011400232 | 0.629345743 | down | 0.03359559 | 1.325358852 | up |
| P09242 | Alpl | Alkaline phosphatase, tissue-nonspecific isozyme OS=Mus musculus OX=10090 GN=Alpl PE=1 SV=2 | 4 | 0.000171279 | 0.630781938 | down | 0.005896269 | 1.343518114 | up |
| Q3TVA9 | Ccdc136 | Coiled-coil domain-containing protein 136 OS=Mus musculus OX=10090 GN=Ccdc136 PE=1 SV=2 | 1 | 0.00230448 | 0.633972603 | down | 0.049072161 | 1.312013829 | up |
| Q8CFT2 | Setd1b | Histone-lysine N-methyltransferase SETD1B OS=Mus musculus OX=10090 GN=Setd1b PE=1 SV=2 | 1 | 0.002426438 | 0.635597979 | down | 0.045512814 | 1.401501767 | up |
| Q9JLB9 | Nectin3 | Nectin-3 OS=Mus musculus OX=10090 GN=Nectin3 PE=1 SV=1 | 1 | 3.97859E-07 | 0.643397813 | down | 0.006627681 | 1.366448802 | up |
| Q8BFP9 | Pdk1 | [Pyruvate dehydrogenase (acetyl-transferring)] kinase isozyme 1, mitochondrial OS=Mus musculus OX=10090 GN=Pdk1 PE=1 SV=2 | 2 | 0.000845009 | 0.652683193 | down | 0.025753944 | 1.224123539 | up |
| Q3UGY8 | Arfgef3 | Brefeldin A-inhibited guanine nucleotide-exchange protein 3 OS=Mus musculus OX=10090 GN=Arfgef3 PE=1 SV=1 | 1 | 0.002039067 | 0.660415465 | down | 0.041643349 | 1.417212757 | up |
| Q91X97 | Ncald | Neurocalcin-delta OS=Mus musculus OX=10090 GN=Ncald PE=1 SV=4 | 7 | 0.00775268 | 0.662890301 | down | 0.045237348 | 1.243017924 | up |
| P11589 | Mup2 | Major urinary protein 2 OS=Mus musculus OX=10090 GN=Mup2 PE=1 SV=1 | 7 | 0.016362305 | 0.665511265 | down | 0.027587006 | 1.403645833 | up |
| Q61234 | Snta1 | Alpha-1-syntrophin OS=Mus musculus OX=10090 GN=Snta1 PE=1 SV=1 | 5 | 4.47472E-05 | 0.665570175 | down | 0.045740355 | 1.204695222 | up |
| P97863 | Nfib | Nuclear factor 1 B-type OS=Mus musculus OX=10090 GN=Nfib PE=1 SV=2 | 4 | 0.00036015 | 0.677282976 | down | 0.040253666 | 1.269662921 | up |
| Q6P9J5 | Kank4 | KN motif and ankyrin repeat domain-containing protein 4 OS=Mus musculus OX=10090 GN=Kank4 PE=1 SV=1 | 4 | 0.000156214 | 0.679389313 | down | 0.004915342 | 1.27340824 | up |
| Q9CRA0 | Art4 | Ecto-ADP-ribosyltransferase 4 OS=Mus musculus OX=10090 GN=Art4 PE=1 SV=1 | 2 | 0.00690553 | 0.679977344 | down | 0.026332928 | 1.277800916 | up |
| P43274 | H1-4 | Histone H1.4 OS=Mus musculus OX=10090 GN=H1-4 PE=1 SV=2 | 17 | 4.75458E-05 | 0.686802176 | down | 0.030580787 | 1.295539808 | up |
| Q8CJ96 | Rassf8 | Ras association domain-containing protein 8 OS=Mus musculus OX=10090 GN=Rassf8 PE=2 SV=1 | 1 | 0.003604003 | 0.686860068 | down | 0.0081659 | 1.271221532 | up |
| Q80UG2 | Plxna4 | Plexin-A4 OS=Mus musculus OX=10090 GN=Plxna4 PE=1 SV=3 | 2 | 0.00062899 | 0.688652879 | down | 0.017379194 | 1.200892857 | up |
| P49766 | Vegfb | Vascular endothelial growth factor B OS=Mus musculus OX=10090 GN=Vegfb PE=2 SV=2 | 1 | 0.005629563 | 0.689169779 | down | 0.004570521 | 1.300125052 | up |
| P51885 | Lum | Lumican OS=Mus musculus OX=10090 GN=Lum PE=1 SV=2 | 9 | 0.006403823 | 0.690275436 | down | 0.037708852 | 1.215798046 | up |
| Q60819 | Il15ra | Interleukin-15 receptor subunit alpha OS=Mus musculus OX=10090 GN=Il15ra PE=1 SV=1 | 1 | 0.016513986 | 0.690496215 | down | 0.006854144 | 1.205846529 | up |
| P09813 | Apoa2 | Apolipoprotein A-II OS=Mus musculus OX=10090 GN=Apoa2 PE=1 SV=2 | 1 | 0.04442644 | 0.692083818 | down | 0.016798626 | 1.339360807 | up |
| P97927 | Lama4 | Laminin subunit alpha-4 OS=Mus musculus OX=10090 GN=Lama4 PE=1 SV=2 | 35 | 0.000162485 | 0.692175899 | down | 0.028519745 | 1.268151815 | up |
| Q8BVI5 | Stx16 | Syntaxin-16 OS=Mus musculus OX=10090 GN=Stx16 PE=1 SV=3 | 2 | 0.039394108 | 0.693592469 | down | 0.034020185 | 1.498248687 | up |
| P49813 | Tmod1 | Tropomodulin-1 OS=Mus musculus OX=10090 GN=Tmod1 PE=1 SV=2 | 17 | 0.001985507 | 0.696332101 | down | 0.045463948 | 1.239281339 | up |
| O08967 | Cyth3 | Cytohesin-3 OS=Mus musculus OX=10090 GN=Cyth3 PE=1 SV=1 | 6 | 3.39933E-05 | 0.697952218 | down | 0.04083771 | 1.235126324 | up |
| Q9JHU2 | Palmd | Palmdelphin OS=Mus musculus OX=10090 GN=Palmd PE=1 SV=1 | 5 | 0.000837787 | 0.698723404 | down | 0.023884313 | 1.222898904 | up |
| P56857 | Cldn18 | Claudin-18 OS=Mus musculus OX=10090 GN=Cldn18 PE=1 SV=1 | 6 | 0.000947547 | 0.704159344 | down | 0.046518291 | 1.324043261 | up |
| Q9Z0P4 | Palm | Paralemmin-1 OS=Mus musculus OX=10090 GN=Palm PE=1 SV=1 | 11 | 0.000791672 | 0.70705347 | down | 0.043085526 | 1.205953339 | up |
| Q8BZF8 | Pgm5 | Phosphoglucomutase-like protein 5 OS=Mus musculus OX=10090 GN=Pgm5 PE=1 SV=2 | 14 | 0.000918163 | 0.710352739 | down | 0.024613304 | 1.225272507 | up |
| P02468 | Lamc1 | Laminin subunit gamma-1 OS=Mus musculus OX=10090 GN=Lamc1 PE=1 SV=2 | 50 | 0.001505015 | 0.710734133 | down | 0.004054034 | 1.296707819 | up |
| P28653 | Bgn | Biglycan OS=Mus musculus OX=10090 GN=Bgn PE=1 SV=1 | 10 | 0.007154566 | 0.713035871 | down | 0.044520187 | 1.279345603 | up |
| Q8QZT4 | Crb3 | Protein crumbs homolog 3 OS=Mus musculus OX=10090 GN=Crb3 PE=1 SV=1 | 1 | 0.000136963 | 0.713220533 | down | 0.039460867 | 1.21672698 | up |
| Q62165 | Dag1 | Dystroglycan OS=Mus musculus OX=10090 GN=Dag1 PE=1 SV=4 | 12 | 0.000166522 | 0.715442452 | down | 0.037041899 | 1.240097009 | up |
| P50592 | Tnfsf10 | Tumor necrosis factor ligand superfamily member 10 OS=Mus musculus OX=10090 GN=Tnfsf10 PE=2 SV=1 | 2 | 0.023669118 | 0.716656608 | down | 0.014069524 | 1.394526316 | up |
| P11531 | Dmd | Dystrophin OS=Mus musculus OX=10090 GN=Dmd PE=1 SV=3 | 16 | 0.001312296 | 0.717165899 | down | 0.010517679 | 1.219678715 | up |
| P18608 | Hmgn1 | Non-histone chromosomal protein HMG-14 OS=Mus musculus OX=10090 GN=Hmgn1 PE=1 SV=2 | 2 | 0.00072837 | 0.722723305 | down | 0.013870877 | 1.239533011 | up |
| Q8C033 | Arhgef10 | Rho guanine nucleotide exchange factor 10 OS=Mus musculus OX=10090 GN=Arhgef10 PE=1 SV=2 | 3 | 0.003341328 | 0.727088754 | down | 0.047049508 | 1.203180915 | up |
| P21279 | Gnaq | Guanine nucleotide-binding protein G(q) subunit alpha OS=Mus musculus OX=10090 GN=Gnaq PE=1 SV=4 | 13 | 0.00025825 | 0.729209221 | down | 0.048643085 | 1.230492197 | up |
| Q61001 | Lama5 | Laminin subunit alpha-5 OS=Mus musculus OX=10090 GN=Lama5 PE=1 SV=4 | 25 | 0.000111454 | 0.731109799 | down | 0.015808745 | 1.26524021 | up |
| P02469 | Lamb1 | Laminin subunit beta-1 OS=Mus musculus OX=10090 GN=Lamb1 PE=1 SV=3 | 43 | 0.001057215 | 0.73215859 | down | 0.029997279 | 1.244283995 | up |
| Q64471 | Gstt1 | Glutathione S-transferase theta-1 OS=Mus musculus OX=10090 GN=Gstt1 PE=1 SV=4 | 10 | 2.78835E-05 | 0.732864675 | down | 0.046606122 | 1.233013589 | up |
| Q9WVH9 | Fbln5 | Fibulin-5 OS=Mus musculus OX=10090 GN=Fbln5 PE=1 SV=1 | 8 | 0.008149478 | 0.732864675 | down | 0.007069617 | 1.231814548 | up |
| Q8K2A1 | Gulp1 | PTB domain-containing engulfment adapter protein 1 OS=Mus musculus OX=10090 GN=Gulp1 PE=1 SV=1 | 6 | 0.000385538 | 0.733605363 | down | 0.011108029 | 1.212554629 | up |
| Q8VCC9 | Spon1 | Spondin-1 OS=Mus musculus OX=10090 GN=Spon1 PE=1 SV=1 | 19 | 0.000347459 | 0.73514632 | down | 0.045420323 | 1.258946522 | up |
| Q02858 | Tek | Angiopoietin-1 receptor OS=Mus musculus OX=10090 GN=Tek PE=1 SV=2 | 7 | 0.00028337 | 0.739028672 | down | 0.04391408 | 1.209422011 | up |
| Q06335 | Aplp2 | Amyloid-like protein 2 OS=Mus musculus OX=10090 GN=Aplp2 PE=1 SV=4 | 6 | 8.0891E-05 | 0.740404336 | down | 0.039602588 | 1.210922042 | up |
| Q9D3N8 | Grtp1 | Growth hormone-regulated TBC protein 1 OS=Mus musculus OX=10090 GN=Grtp1 PE=1 SV=1 | 1 | 0.003375318 | 0.740424278 | down | 0.017243885 | 1.230799841 | up |
| Q8BW75 | Maob | Amine oxidase [flavin-containing] B OS=Mus musculus OX=10090 GN=Maob PE=1 SV=4 | 15 | 0.00039299 | 0.741121221 | down | 0.03673003 | 1.214653465 | up |
| Q61789 | Lama3 | Laminin subunit alpha-3 OS=Mus musculus OX=10090 GN=Lama3 PE=1 SV=4 | 24 | 0.003945895 | 0.741183503 | down | 0.013614855 | 1.27983871 | up |
| Q9JL18 | Bace2 | Beta-secretase 2 OS=Mus musculus OX=10090 GN=Bace2 PE=1 SV=1 | 1 | 0.015370699 | 0.741394792 | down | 0.001877745 | 1.28461849 | up |
| P08122 | Col4a2 | Collagen alpha-2(IV) chain OS=Mus musculus OX=10090 GN=Col4a2 PE=1 SV=4 | 7 | 0.016320669 | 0.745978756 | down | 0.001749432 | 1.321399512 | up |
| Q8K190 | Saysd1 | SAYSvFN domain-containing protein 1 OS=Mus musculus OX=10090 GN=Saysd1 PE=1 SV=1 | 1 | 0.002232612 | 0.751404079 | down | 0.009992389 | 1.209677419 | up |
| P15306 | Thbd | Thrombomodulin OS=Mus musculus OX=10090 GN=Thbd PE=1 SV=1 | 11 | 0.044756556 | 0.751481043 | down | 0.008870323 | 1.216791486 | up |
| Q2KN98 | Specc1l | Cytospin-A OS=Mus musculus OX=10090 GN=Specc1l PE=1 SV=1 | 20 | 0.004877058 | 0.752815649 | down | 0.039428118 | 1.215354331 | up |
| E9Q414 | Apob | Apolipoprotein B-100 OS=Mus musculus OX=10090 GN=Apob PE=1 SV=1 | 12 | 0.047094466 | 0.753351206 | down | 0.03299482 | 1.231316726 | up |
| Q80VP0 | Tecpr1 | Tectonin beta-propeller repeat-containing protein 1 OS=Mus musculus OX=10090 GN=Tecpr1 PE=1 SV=1 | 7 | 0.000189158 | 0.763888889 | down | 0.006585461 | 1.248221344 | up |
| B2B9E1 | Triqk | Triple QxxK/R motif-containing protein OS=Mus musculus OX=10090 GN=Triqk PE=2 SV=1 | 1 | 0.006480114 | 0.765691651 | down | 0.021128083 | 1.275368086 | up |
| Q61292 | Lamb2 | Laminin subunit beta-2 OS=Mus musculus OX=10090 GN=Lamb2 PE=1 SV=2 | 39 | 0.000926727 | 0.76686747 | down | 0.002597926 | 1.230164965 | up |
| Q3V3R4 | Itga1 | Integrin alpha-1 OS=Mus musculus OX=10090 GN=Itga1 PE=1 SV=2 | 30 | 0.000332708 | 0.768676868 | down | 0.049583875 | 1.211553474 | up |
| Q14B46 | Rtkn2 | Rhotekin-2 OS=Mus musculus OX=10090 GN=Rtkn2 PE=1 SV=2 | 8 | 0.002922183 | 0.770432692 | down | 0.012151857 | 1.211778471 | up |
| Q8BY89 | Slc44a2 | Choline transporter-like protein 2 OS=Mus musculus OX=10090 GN=Slc44a2 PE=1 SV=2 | 9 | 0.015716493 | 0.772478736 | down | 0.048708498 | 1.244986237 | up |
| Q80VQ1 | Lrrc1 | Leucine-rich repeat-containing protein 1 OS=Mus musculus OX=10090 GN=Lrrc1 PE=1 SV=2 | 6 | 0.002310131 | 0.776902092 | down | 0.016631133 | 1.223956301 | up |
| Q8BRV5 | Kiaa1671 | Uncharacterized protein KIAA1671 OS=Mus musculus OX=10090 GN=Kiaa1671 PE=2 SV=1 | 2 | 0.029387107 | 0.786065071 | down | 0.047121352 | 1.242483405 | up |
| Q4LFA9 | Sema3g | Semaphorin-3G OS=Mus musculus OX=10090 GN=Sema3g PE=2 SV=1 | 3 | 0.010394684 | 0.788625304 | down | 0.005572757 | 1.202853837 | up |
| Q9D0J8 | Ptms | Parathymosin OS=Mus musculus OX=10090 GN=Ptms PE=1 SV=3 | 4 | 0.01243867 | 0.793514836 | down | 0.018885449 | 1.209329221 | up |
| Q8CFA2 | Amt | Aminomethyltransferase, mitochondrial OS=Mus musculus OX=10090 GN=Amt PE=1 SV=1 | 1 | 0.000579255 | 0.799938157 | down | 0.003779242 | 1.22883649 | up |
| Q61092 | Lamc2 | Laminin subunit gamma-2 OS=Mus musculus OX=10090 GN=Lamc2 PE=1 SV=2 | 12 | 0.015300713 | 0.80068408 | down | 0.014687913 | 1.246990291 | up |
| Q8C790 | Fam221a | Protein FAM221A OS=Mus musculus OX=10090 GN=Fam221a PE=1 SV=1 | 1 | 0.007647923 | 0.80361596 | down | 0.034618495 | 1.24670287 | up |
| Q91VA3 | Capn8 | Calpain-8 OS=Mus musculus OX=10090 GN=Capn8 PE=1 SV=1 | 1 | 0.009593726 | 0.806145074 | down | 0.049205088 | 1.295874263 | up |
| P40240 | Cd9 | CD9 antigen OS=Mus musculus OX=10090 GN=Cd9 PE=1 SV=2 | 5 | 0.00033644 | 0.807716241 | down | 0.013722974 | 1.229198767 | up |
| Q91V88 | Npnt | Nephronectin OS=Mus musculus OX=10090 GN=Npnt PE=1 SV=1 | 11 | 0.004038069 | 0.809110629 | down | 0.006010542 | 1.211030257 | up |
| Q61087 | Lamb3 | Laminin subunit beta-3 OS=Mus musculus OX=10090 GN=Lamb3 PE=1 SV=2 | 17 | 9.50612E-05 | 0.81073534 | down | 0.001666479 | 1.210868733 | up |
| Q8CB27 | Yod1 | Ubiquitin thioesterase OTU1 OS=Mus musculus OX=10090 GN=Yod1 PE=1 SV=1 | 1 | 0.017459433 | 0.813580635 | down | 0.049393307 | 1.248454405 | up |
| B2RX12 | Abcc3 | ATP-binding cassette sub-family C member 3 OS=Mus musculus OX=10090 GN=Abcc3 PE=1 SV=2 | 2 | 0.021639992 | 0.819379116 | down | 0.011423432 | 1.223497895 | up |
| Q80V42 | Cpm | Carboxypeptidase M OS=Mus musculus OX=10090 GN=Cpm PE=1 SV=2 | 3 | 0.026391774 | 0.829848826 | down | 0.010489938 | 1.283333333 | up |
| Orange and green indicated upregulated or downregulated expression. | | | | | | | | | |
